# Do Dogs Provide Information Helpfully?

**DOI:** 10.1371/journal.pone.0159797

**Published:** 2016-08-10

**Authors:** Patrizia Piotti, Juliane Kaminski

**Affiliations:** Centre for Comparative and Evolutionary Psychology, Department of Psychology, University of Portsmouth, Portsmouth, Hampshire, United Kingdom; University of York, UNITED KINGDOM

## Abstract

Dogs are particularly skilful during communicative interactions with humans. Dogs’ abilities to use human communicative cues in cooperative contexts outcompete those of other species, and might be the result of selection pressures during domestication. Dogs also produce signals to direct the attention of humans towards outside entities, a behaviour often referred to as *showing behaviour*. This *showing behaviour* in dogs is thought to be something dogs use intentionally and referentially. However, there is currently no evidence that dogs communicate helpfully, i.e. to inform an ignorant human about a target that is of interest to the human but not to the dog. Communicating with a helpful motive is particularly interesting because it might suggest that dogs understand the human’s goals and need for information. In study 1, we assessed whether dogs would abandon an object that they find interesting in favour of an object useful for their human partner, a random novel distractor, or an empty container. Results showed that it was mainly self-interest that was driving the dogs’ behaviour. The dogs mainly directed their behaviour towards the object they had an interest in, but dogs were more persistent when *showing* the object relevant to the human, suggesting that to some extent they took the humans interest into account. Another possibility is that dogs’ behaviour was driven by an egocentric motivation to interact with novel targets and that the dogs’ neophila might have masked their helpful tendencies. Therefore, in study 2 the dogs had initial access to both objects, and were expected to indicate only one (relevant or distractor). The human partner interacted with the dog using vocal communication in half of the trials, and remaining silent in the other half. Dogs from both experimental groups, i.e. indicating the relevant object or indicating the distractor, established joint attention with the human. However, the human’s vocal communication and the presence of the object relevant to the human increased the persistency of *showing*, supporting the hypothesis that the dogs understood the objects’ relevance to the human. We propose two non-exclusive explanations. These results might suggest that informative motives could possibly underlie dogs’ *showing*. It is also possible that dogs might have indicated the location of the hidden object because they recognised it as the target of the human’s search. This would be consistent with taking into account the objects’ relevance, without necessarily implying that the dogs understood the human’s state of knowledge.

## Introduction

Dogs are particularly good at understanding human communication, for example they can find hidden food following communicative cues provided by humans [1–3]. This was demonstrated in a series of studies using the so-called *object-choice task*. In this task a piece of reward is hidden underneath one of several containers, and afterwards a human indicates the correct container to the dog by e.g. pointing at it [1,4,5]. Dogs demonstrated to be extremely skilful in following this gesture both from a very young age and without the need for any explicit training [4,6–8]. When compared to their closest living relative, the wolf, dogs performed better even when both species were raised under identical conditions [7,9,10] unless wolves received extensive and prolonged training [6,11].

The reasons for dogs’ outstanding abilities in inter-specific communication with humans are thought to depend on dogs’ unique evolutionary history [7,12]. Dogs are the most ancient domesticated species [13–15] and it has been hypothesised that humans bred them selectively for certain activities, such as hunting and herding [16], where it was important for dogs to be particularly skilful at following human communication [17]. One hypothesis is therefore that, as an adaptation to life with humans, dogs developed specific socio-communicative skills for interacting with humans [1,7,12,18].

Dogs seem to be flexible not only in how they use communicative signals coming from humans but also in their production of communicative behaviours towards humans, such as the one described as *showing behaviour* [4,19]. The term *showing behaviour* summarises actions like gaze alternation and other communicative signals through which dogs indicate a hidden object or food to a human [19]. There is evidence that *showing behaviour* fulfils all the criteria required for identifying intentionality and referentiality as they had been introduced for primates [20,21]. Specifically, dogs do not indicate in the absence of an audience, they alternate gazes between the human and the referent, they use attention getting behaviours (e.g. vocalisations) [19] they take into account the attentional state of their audience [22,23], and finally they show persistence and elaboration when their communication is not successful [24].

Dogs’ flexible use of inter-specific communication with humans raises researchers’ interest in the cognitive mechanisms underlying such skills. One question that is currently understudied is to what extent dogs communicate to truly *inform* a human partner about the hidden object. In the infant literature, the informative intent [25,26] is described as a subtype of *declarative* communication (i.e. communicating to share an experience or influence someone’s mental state), as opposed to *imperative* communication (i.e. communicating to obtain an object or influence someone’s behaviour) [27–29]. Some consider human communication to rely on mechanisms unique to humans [30–32]. One is the presence of a common ground, i.e. a body of knowledge, beliefs and suppositions that two speakers believe they share with each other [33,34]. Forming a common ground with another individual might require to some extent the ability to make inferences about the other individual’s mental states. The other is a unique cooperative tendency, which humans expect when they communicate [32]. Some authors consider these to be uniquely human traits and the reason why humans, from a very young age, can successfully infer the location of a hidden toy from following an adults pointing gesture, while humans’ closest relatives, the chimpanzees, fail to do so [35]. Children also produce pointing helpfully to inform others about the location of a relevant object without expecting anything in return, as opposed to chimpanzees, who would not produce pointing gestures unless there is something in it for them [25,36].

However, other authors have challenged the idea that declarative pointing requires the understanding of another individual’s mental state or goals, or the presence of a common ground, and argue for explanations of preverbal human communication that do not require the understanding of internal state [20,21,37–39]. Gergely and Csibra suggest two mechanisms that do not require the understanding of mental states. The first mechanism suggests that children understand actions, including communication, in a *referential* and *teleological* way, i.e. they can link others’ behaviour to a certain object, and they interpret actions as directed to a certain goal [40–43]. The second mechanism implies that human communication relies on “*natural pedagogy*”, i.e. it is characterised by a series of elements that allow and facilitate the transfer of knowledge. Specifically, humans, from a very young age, are sensitive to ostensive cues indicating that they are addressed in the communication, have referential expectations after observing ostensive cues, and interpret ostensive-referential communication as conveying information that is relevant and generalizable [43,44]. Similar mechanisms are thought to be possible, to a certain degree, in non-human animals [38,40,44,45], including dogs [46–48].

Kaminski and colleagues [49] tested whether dogs produce informative communicative behaviours by confronting dogs with a situation during which the humans and the dogs’ motivation to receive the hidden object varied. They showed that dogs indicate the location of a hidden object to a human if the dogs had a selfish interest in the hidden object, but not if only the human had an interest in it. Humans’ and dogs’ interest in the object was determined by the context and by who interacted with the object before it was hidden. Either only the dog interacted with the object (e.g. a dog toy), or the human and the dog interacted with the object, or only the human interacted with the object. Afterwards a second person hid the object while the first person left the room. The first person then returned and asked the dog to find the object. Dogs communicated the location reliably only if they had an interest in the hidden object. In a follow up study, two objects were hidden at the same time. One was an object that the human had an interest in and the dog had seen the human use, while the other was a distractor object that the human ignored entirely. In this case, the dogs did not distinguish between the two objects. This result suggests that either dogs do not have the motivation to attend to the humans needs, or lack the cognitive capacity to understand the humans’ lack of knowledge and need for information [49].

Kaminski and colleagues’ study suggests that there is of yet no evidence that dogs understand the informative element of communication [49] despite their unique skills in communicating with humans [50]. Indeed, dogs could possibly interpret human communication (e.g. pointing) as an imperative, i.e. the human is directing them on where to go [32] or what to do [49,51]. In this scenario dogs would also produce their communicative behaviours towards humans without any intent of influencing the humans’ state of mind. If dogs’ communication were either a request or a response to a command to fetch, they would be communicating without necessarily understanding others’ state of knowledge and goals [52]. However, the study by Kaminski and colleagues could not tease apart the possibilities that the dogs’ behaviour was dues to a lack of helpful motivation, or due to their inability to understand the need for information and the relevance of the object for the human partner [49].

The current study therefore aims to further investigate dogs’ collaborative and informative motives during communication. We also aimed at assessing dogs’ ability to understand an object’s relevance after they see a human partner using it. In study 1, we examined whether dogs would abandon a hidden dog toy to indicate the location of another object that a human partner wanted. It is possible that the objects’ novelty and the humans’ requests, rather than relevance, influenced the dogs’ choices in such situation. Therefore, in study 2 we examined whether dogs are able to understand that the human partner wanted an object that she had previously used, over a distractor that she had previously ignored. If dogs are driven to use the *showing behaviour* based on an informative intent, then we would expect the dogs to *show* prevalently the object relevant to the human over a distractor, as suggested by previous research in infants [25,26]. On the contrary, if the motivation underlying dogs’ communication is to request, or an attempt to respond to a human's command to fetch, as the results by Kaminski et al. would suggest [49] then we would expect dogs to either indicate only objects that they have an interest in or indicate equally any hidden object, without differentiate based on the object's relevance to the human partner.

The studies were carried out in strict accordance with the recommendations in the ASAB/ABS guidelines for the use of animals in research and were approved by the University of Portsmouth Animal Ethics Committee. Dog owners were informed about the procedure involved and gave their permission for their dog to participate in the study.

## Study 1

The general procedure of this study was modelled on the study designed by Kaminski and colleagues [49]. Dogs knew the location of a hidden dog toy and the content of a second hiding place (i.e. an object *relevant* for the human, an object *useless* for the human, or *no object*); we wanted to know if dogs would indicate the location of an object depending on the human’s interest in the object. It was hypothesised that abandoning the dog toy in favour of indicating the *relevant object* suggested a motivation to help. More consistent indications towards the *relevant object*, rather than the other useless object (a *distractor*), would also indicate that dogs understood the objects’ relevance for the experimenter.

### Subjects

A sample of 29 adult dogs was recruited for this study. Four dogs had to be excluded from testing because they did not settle during the warm-up, and one dog was tested but excluded from subsequent analysis because of a procedural mistake. Dogs were recruited through the Dog Cognition Centre Portsmouth Register and through contacts with local dog training groups. The inclusion criteria for the study were that dogs had to be between 1 and 10 years old and had to be comfortable and relaxed while being separated from their owner for the duration of the test. In addition, the dogs had to be toy motivated. All dogs were normal family dogs that lived with their owners and had the training background typical for a pet dog. Some of the dogs had participated in other studies before, but not studies using an experimental paradigm similar to the one used here.

Twenty-four dogs, 16 males and 8 females, represented the final sample (S1 Dataset). Twelve dogs were crossbreeds and twelve were pure breeds (according to the *British Kennel Club Breed Groups*, as defined by the British Kennel Club. these consisted of 6 *Gundogs*, 1 *Hound*, 1 *Pastoral*, 2 *Terriers*, 1 *Working*, 1 *Utility*). The age of the dogs ranged between 1.5 and 8 years (M = 3.8, SD = 1.7).

## Methods

**Fig 1:**
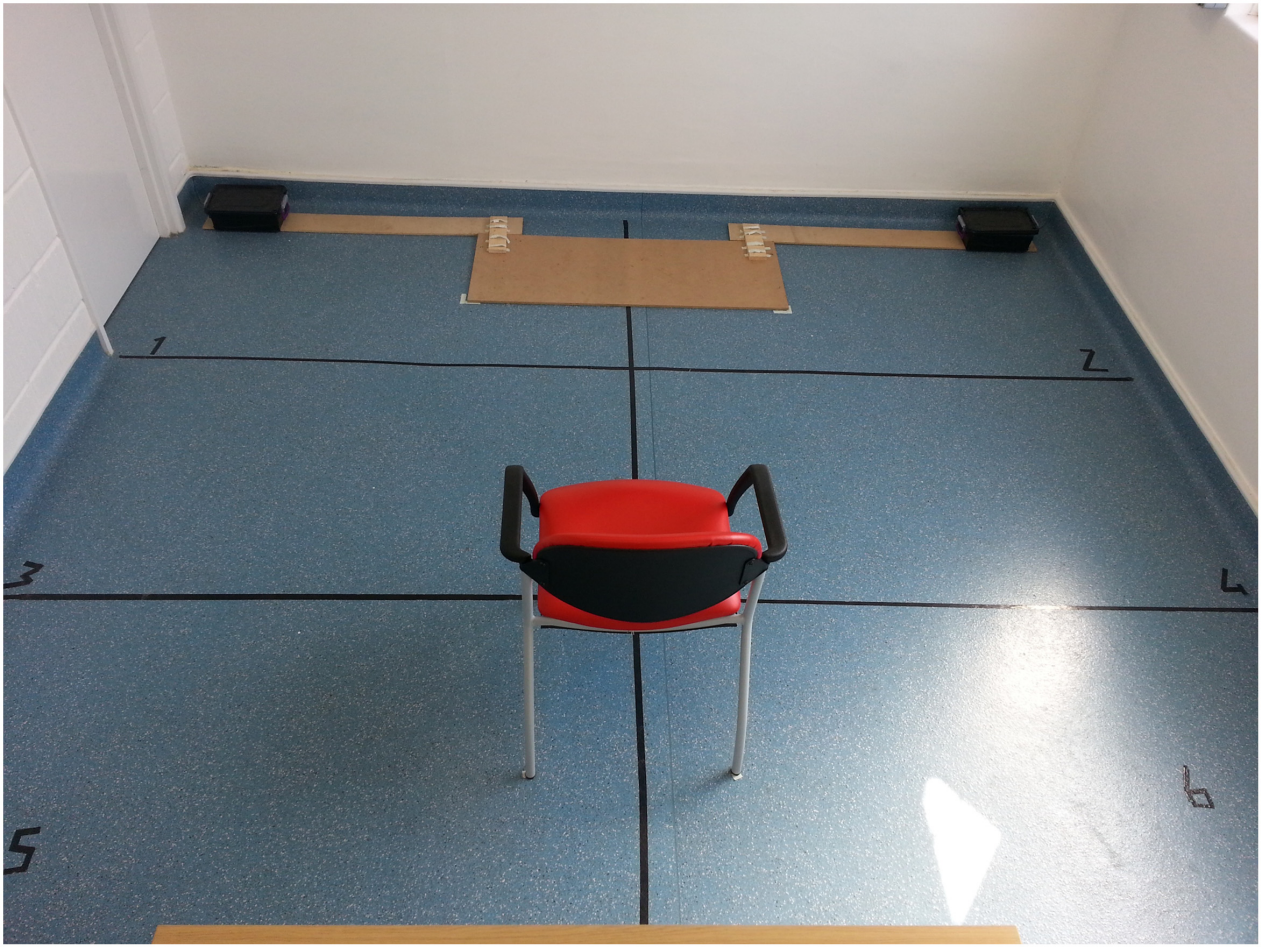
Testing room for study 1. A chair was placed in the testing room for the experimenter to sit on. Two opaque containers were positioned in front of the chair at the two corners of the room, so that the chair was equidistant from each container.

Testing took place in one of the rooms (3.70 m x 4.20 m) of the Dog Cognition Centre Portsmouth (DOCS). Two opaque containers (19 cm x 10 cm) were placed on the floor, one in the left and the other in the right corner of the room. A chair for the experimenter to sit on was placed equidistant to both containers (Fig 1). Different objects were used as hidden targets: a notepad, stapler or a dog toy.

### Procedure

In order to allow the dogs to habituate with the environment and with the people involved, the dogs were first allowed to explore the experimental room. During this time both the experimenter and the helper interacted with the dog to ensure the dog was familiar with them, while avoiding playful interactions with the dog in an attempt to not create a play context for the dogs, which might have affected the study.

After this warm up the experimenter sat down on the chair provided and started writing notes, using the notepad (“*relevant object*”). The helper stood about a meter away from her while the dog was allowed to roam around freely. To ensure that the dog attended to the experimenter’s activity, the experimenter and the helper now and then called the dog’s attention and encouraged the dog to stay near them while avoiding indicating the notepad specifically at any time. During this demonstration, only the *relevant object* was in the room; the *dog toy* and the *distractor* were left outside and out of the view of the dog. The rationale behind this set up was to prevent dogs from being distracted by the other objects during the demonstration. At the end of the demonstration, the experimenter left the room and took the *relevant object* with her, placed it with the others in a container outside the room, and walked away. The set up therefore ensured that all objects were already out of the room before the hiding phase. This allowed the helper to take the objects to be hidden, while avoiding the experimenter seeing them.

Each dog was presented with 6 trials (two per condition: *relevant*, *distractor*, and *no object*) and each trial consisted of a demonstration, followed by a searching phase (described below). The dog was given a few minutes break at the end of each searching phase, before starting another trial, while the helper set up the room for the following trial. The demonstration in trial 1 lasted about 40 seconds, whilst demonstrations in trials 2–6 were reduced to about 20 seconds in order to prevent the dogs from losing interest. The order with which the demonstrations were administered was counterbalanced across dogs, so that each condition was presented in the first trial (with the longer demonstration) for a third of the dogs. After this time elapsed the experimenter left the room through door A (Fig 1) together with the helper. The helper then returned and, depending on the condition, hid one or two objects in the boxes provided.

“*Relevant*” condition: The helper returned to the room, holding the *dog toy* and the *relevant object* (notepad) in her hands. While ensuring that the dog was watching, the helper hid the *dog toy* in one container and the *relevant object* in the other container.
*“Distractor”* condition: The helper returned to the room holding a *dog toy* and the *distractor* (stapler) in her hands. While ensuring that the dog was watching, the helper hid the *dog toy* in one container and the *distractor* in the other container.
*“No object”* condition (baseline): The helper returned to the room holding only a *dog toy* in her hands. While ensuring that the dog was watching, the helper hid the *dog toy* in one of the two containers and showed the dog that the other container was empty.

The helper always baited the containers starting with the left one first. The location of objects was counterbalanced and semi-randomised across trials and conditions with the stipulation that the same type of object could not be in the same location in more than two consecutive trials. During the hiding phase the helper made sure the dog could see closely the objects that were hidden so that the dogs could recognise the object that they had observed earlier during the demonstration.

After the hiding was completed the helper left the testing room, cueing the experimenter to enter. The experimenter held a pen in her hand in an attempt to indicate that she was going to continue her previous activity. The experimenter then started searching the area around the chair for a few seconds as if she was looking for the notepad, which she needed for her activity. Upon not finding it, she sat on the chair and followed a pre-determined script, similar to that of Kaminski and colleagues [49], where the duration of each phase was determined using a timer:

*Phase 1*—the experimenter searched for the object for 20 s while performing the following activities: repeatedly lifting her arms and shoulders and saying ‘Hmm, that’s weird. It was there, and now it’s gone. I don’t understand.’ and repeatedly mentioning the dog’s name. In order to prevent influencing the dog by gazing at the containers, the researcher kept her gaze on the dog the entire time, as in Viranyi and colleagues’ procedure [53]. While doing so, she remained seated the entire time.
*Phase 2*—the experimenter started formulating more specific questions which were directed at the dog, ‘Where is it? Where has it gone?’, for 20 s while producing the same arm and shoulder movements, and repeatedly mentioning the dog’s name. Again, she looked only at the dog and remained seated.
*Phase 3*—the experimenter stood up while remaining silent for a few seconds and continued to look at the dog.
*Phase 4*—the experimenter tried to guess the location of the notepad based on the dogs’ behaviour and made a decision. If the experimenter found the notepad, she retrieved it saying ‘Wow, there it is! Great!’, and put it in her pocket without offering it to the dog or praising the dog in any way. If she did not find the notepad in the container that she opened, she closed the container without touching the content and saying ‘Oh, too bad! It’s not here’. If the experimenter could not infer where the object could be based on the dog’s behaviour, she just lifted her arms and shoulders saying ‘Too bad, we can’t find it’. Although the phrasing changed, the tone of the experimenter’s voice and her expressions were kept as similar as possible in all cases. After each of these possible events the trial was over; the experimenter took the dog out through door B, while the helper returned to the testing room and re-set the room for the next trial.

The overall design was a within subjects design where all dogs participated in all conditions and received 2 trials per condition summing up to 6 trials altogether. Trials were presented blocked by condition with the order of conditions counterbalanced across subjects.

### Behaviour analysis

Digital video footage was taken from all trials and the Solomon Coder software (beta 091110, copyright 2006–2008 by András Péter, developed at ELTE TTK Department of Ethology, Budapest, Hungary) was used to record dogs’ behaviour during testing. The software was set up with a sensitivity of .10 seconds.

The direction of gazing in the search phase was recorded on the basis of the orientation of the head of the dog. The frequency and duration of gazing toward three distinctive locations in the room was recorded: (1) gazing at the experimenter, (2) gazing at the box where the *dog toy* was hidden, (3) gazing at the *target box* (i.e. the other box). Gazes were also subjected to a sequential analysis. According to the definition of “gaze alternation” by Miklósi and colleagues [19], a gazing sequence consisting of two gazing units was recorded when gazing at the experimenter was followed directly by a gaze at one of the two boxes within 2 seconds or vice versa. Specifically, coders followed the rule that there could be a maximum gap of 2 seconds between the end of the first gaze in the alternation and the beginning of the following one. For example, if the dog looked at the box first and then at the experimenter, there could be no more than 2 seconds between the end of the look to the box and the beginning of the look to the experimenter.

Finally, the first hiding place that dogs indicated in the search phase (with their position, orientation of the body or orientation of the head) was recorded.

Since dogs’ level of attention during the demonstration might vary, we also recorded the amount of time that dogs spent looking at the experimenter during the demonstration, i.e. the overall duration of looks to the experimenter in this phase. Looking was defined as the dogs head being oriented toward the experimenter and was recorded from the moment the experimenter started writing on the note-pad, to the moment she stood up to leave the room.

A random selection of the video material (20%) was coded by a second observer, naïve to the purpose of the study and to the content of the hiding boxes. The correlation between the two coders was calculated using Spearman r, and inter-coder reliability was assessed according to the limits given by Landis & Koch [54].

Inter-observer reliability was substantial for the frequency of gazes to the *dog toy* (r_s_ = .78, N = 28, p = .001), the frequency of gazes to the *target box* (r_s_ = .65, N = 28, p = .001), the duration of gazes to the *target box* (r_s_ = .72, N = 28, p = .001), and the gaze alternations between the experimenter and the *target box* (r_s_ = .75, N = 28, p = .001). There was an excellent agreement on the duration of gazes to the *dog toy* (r_s_ = .88, N = 28, p = .001), the frequency of gaze alternations between the experimenter and the *dog toy* (r_s_ = .80, N = 28, p = .001), and the duration of gazes during the demonstration (r_s_ = .82, N = 30, p = .001).

### Statistical analysis

Data were analysed using the statistical software R [55], with the packages *lme4* [56], *MuMIn* [57], and *lsmeans* [58]. A series of generalised linear mixed models (GLMM), fit by maximum likelihood (Laplace Approximation), were calculated for the variables measured. Models were first evaluated through an automated model selection process that generated a set of models with combinations of factors from a global model (which included all the effects in question), ranked them and obtained model weights using the Second-order Akaike Information Criterion (AIC) [59]. The models with lowest AIC were evaluated with a likelihood ratio test against the corresponding null models (i.e. including only control factors). If the comparison was significant then Laplace estimated *p*-values were calculated for the different fixed effects of the model with lowest AIC [60]. Pairwise post-hoc comparisons were obtained from a Tukey test in the absence of interactions, while the least-squares of means method was used in case of interaction between categorical factors. If there was a significant interaction between fixed factors, only *p*-values for the interaction effects will be reported because the significance of main effects is uninterpretable in case of a significant interaction [61]. All results have been reported with standard errors.

A GLMM (null model) with logit function was calculated with the binary response variable “indication of the target” (yes, no), and the nested random intercept factors “dog”, “trial” and “toy side” (N = 144, number of subjects = 24). All the relevant fixed factors and interactions were included in the model (S1 Text for details). The model that yielded the lowest AIC comprised the fixed factors “condition” and “attention during demonstration”, without interaction.

A GLMM (null model) with log function was calculated with the response variable “frequency of gaze alternations” and the fixed factor “direction of the gaze alternation” (toy-box, target-box). The likelihood ratio test showed that the null model with a dog-specific slope for the factor “direction of the gaze alternation” yielded a significantly lower AIC. Therefore the nested random slope factors “dog”, “trial” and “toy side” (N = 144, number of subjects = 24) were included in the null model. All the relevant fixed factors and interactions were included in the model (S1 Text for details). The model that yielded the lowest AIC comprised the fixed factors “direction of the gaze alternation” and “trial”, without interaction.

The last GLMM (null model) with logit function was calculated with the response variable “duration of gazes (s)” weighted by the factor “duration of the trial (s)” and the fixed factor “direction of the gaze” (experimenter, toy-box, target-box, other). All the relevant fixed factors and interactions were included in the model (S1 Text for details). The nested random intercept factors “dog”, “trial” and “toy side” (N = 144, number of subjects = 24) were included in the model. The model that yielded the lowest AIC comprised the factors “direction”, “condition” (relevant, distractor, no object), and “attention” (s), with a 3 level interaction.

## Results

Overall, dogs first indicated the target on average in 47% of trials. There was a main effect of dogs’ attention during the demonstration and the content of the *target box*, without any interaction, on the number of trials in which the dogs first indicated the *target box* (GLMM_Attention+Condition_, *N* = 24, χ^2^_3_ = 10.679, *p* = 0.013). The probability of indicating the target increased with the time spent looking at the demonstration, with the dogs being more likely to choose the target first in the trials where they were more attentive to the demonstration (estimate _attention_ ± SE = 0.028 ± 0.013, *p* = 0.030). Post-hoc Tukey revealed that when the *relevant object* was in the target box, compared to the *distractor*, dogs were less likely to indicate the target box, though this difference was not significant (estimate _relevant-distractor_ ± SE = − 0.835 ± 0.093, *p* = 0.093). There was also no difference in the dogs’ indications to the target box between the *relevant object* and the *no object* condition (estimate _relevant-no object_ ± SE = − 0.728 ± 0.398, *p* = 0.160), or between the *distractor object* and the *no object* condition (estimate _distractor-no object_ ± SE = 0.1071 ± 0.386, *p* = 0.958).

The analysis of gaze alternations indicated that overall the majority of the dogs alternated their gazes both between the experimenter and the *dog toy* (87%), and between the experimenter the *target box* (75%), (McNemar test: *p* = 0.375). Also, there was no difference in the proportion of dogs that used gaze alternations to indicate the target in the *relevant object* (50%), in the *distractor* condition (67%), and *no object* condition (46%) (Cochran’s Q test: *T* = 3.818, *p* = 0.148).

There was a main effect of the factors “direction of the gaze alternation” and “trial” on the frequency of gaze alternations (GLMM_Direction+Trial_, *N* = 24, χ^2^_1_ = 11.135, *p* = 0.001). The frequency of gaze alternations decreased overall with the progression of trials (estimate _trial_ ± SE = − 0.131 ± 0.039, *p* = 0.001). Post-hoc Tukey test also revealed that dogs were more likely to *show* the toy more often than the target box (estimate _toy—target_ ± SE = 0.731 ± 0.260, *p* = 0.001).

There was a significant effect with a 3 level interaction between the direction of the gaze, condition, and the attention during the demonstration, on the duration of dog gazes (GLMM_Direction*Condition*Attention_, *N* =, χ^2^_27_ = 752.6, *p* = 0.001). Dogs were more likely to gaze longer at the toy box when they were more attentive to the demonstration, both in the *distractor* condition (estimate _toy*distractor*attention_ ± SE = 0.003 ± 0.001, *p* = 0.001) and in the *relevant object* condition (estimate _toy*relevant*attention_ ± SE = 0.002 ± 0.001, *p* = 0.001). However the effect of attention and condition was different when dogs were gazing at the target. In the *distractor* condition, the dogs’ gazes to the target box were shorter when dogs were more attentive to the demonstration (estimate _target*distractor*attention_ ± SE = − 0.002 ± 0.001, *p* = 0.001). On the contrary, in the *relevant object* condition, gazes to the target box were longer when the dogs were more attentive to the demonstration (estimate _target*relevant*attention_ ± SE = 0.003 ± 0.001, *p* = 0.001).

## Discussion

One main finding of this study is that when the dogs paid more attention to the demonstration they were more persistent, i.e. longer, in *showing* the target if it contained the object relevant for the human, rather than a distractor. One possible explanation is that dogs were able to recognise the objects’ relevance based on the demonstration that they witnessed, and that they took that into account when communicating with the experimenter. Such behaviour would be consistent with the definition of informative communication, and comparable to the behaviour of children in similar studies [25].

However it should be noted that the frequency of gaze alternations varied only based on whether the dogs were gazing at the toy or the target box but not the condition (i.e. the target object was *relevant* or a *distractor*). Furthermore, though gaze frequency decreased with trials, the dogs clearly *showed* the toy more often than the target. This suggests that irrespective of condition, dogs could never ignore their own selfish interest for the dog toy in favour of the other objects.

One could argue that the frequency of gazes to the target did not change across conditions because dogs may find it difficult to discriminate across conditions the content of the box that did not contain the toy. It could be that because the objects in the target box are not relevant to dogs, they simply did not differentiate them in their communicative behaviour. Interestingly though the findings show that dogs clearly discriminated the content of the boxes overall and in the different conditions.

Attention also played a role in influencing the behaviour of the dogs. The level of attention during the demonstration affected the persistency of gazes to the target in a way that was consistent with the content’s relevance (i.e. it increased in the *relevant* condition and decreased in the *distractor* condition). This could possibly suggest that attention aided the dogs’ in understanding the relevance of the objects. Another explanation, which does not exclude the previous one, could be that more attentive dogs communicate more. It might be possible that attention to humans increases communication in dogs. Indeed, the number of trials in which the dogs first indicated the target increased with the attention, regardless of the condition. Moreover, gazes to the toy were more persistent when dogs were more attentive in the demonstration.

Finally, the experimenter’s searching behaviour and utterance did not affect the dogs’ overall indications. Dogs are sensitive to ostensive cues in ways very similar to children [62–64], which is something quite unique among non-human species [6]. Cues such as eye contact and high pitch voice appear to help dogs understanding that communication is directed at them [62,63] and help to initiate and maintain communication [42,50,65]. Therefore it would be expected that the human’s high pitch voice would increase dogs’ communication. One possible explanation could be that dogs’ overall orientation used to measure the first indication was not necessarily a communicative behaviour, but rather reflected dogs’ focus of attention. Since dogs were distracted by the presence of the toy and their own interest in it, they did not orientate much towards the target box.

Since it is possible that the dogs’ preference for the dog toy, or the novel object [66] was simply inhibiting their overall behaviour, we conducted a follow up study in which only one object per dog was hidden and it was either an object the human needed or a distractor. Moreover, both objects were in the room and accessible to the dog from the beginning of the trial. The effect of the ostensive cue “high pitch voice” was also investigated systematically. Therefore, for each dog, the experimenter searched for the hidden object in silence for half of the trials, and talked with a high pitch voice in the other half.

## Study 2

In this follow up study dogs witnessed one of two objects being hidden in the room that was either relevant to the experimenter (“*relevant*” group) or was not (“*distractor”* group). The object that was not hidden was taken out of the room by the helper. We also manipulated whether the experimenter used certain ostensive cues (“high pitched voice”) during her search or not.

### Subjects

A sample of 51 dogs was recruited in this study. Dogs were recruited through the Dog Cognition Centre Portsmouth Register and through contacts with local dog training groups. The inclusion criteria for the study were identical to those in study 1. Some of the dogs had participated in other studies before, but not in studies using an experimental paradigm similar to the one used here. None of the dogs had participated in study 1.

Forty-eight dogs took part in this study, 24 dogs per condition (S1 Dataset); an additional dog was recruited but excluded from testing because of aggression and two additional dogs were tested but excluded from analysis because of procedural mistakes. In both groups 17 of the dogs were males and 10 of the dogs were crossbreeds. Pure breed dogs were classified according to the *British Kennel Club Breed Groups*, as defined by the British Kennel Club. In the *relevant* group, the pure breed dogs consisted of: 7 *Gundogs*, 1 *Hound*, 2 *Pastoral dogs*, 1 *Terrier*, 2 *Working dogs*, 1 *Toy*. In the *distractor* group, the pure breed dogs consisted of: 6 *Gundogs*, 2 *Pastoral dogs*, 1 *Terrier*, 3 *Working dogs*, and 2 *Utility*. The age of the dogs ranged between 1 and 10 years in the *relevant* group (M = 4.1, SD = 2.8), and between 1 and 9.5 years in the *distractor* group (M = 4.3, SD = 2.4).

## Methods

The study followed a procedure similar to that of study 1, with the difference that now only one object was hidden in one of three possible locations and that object was either relevant to the experimenter (notepad) or not (stapler).

**Fig 2:**
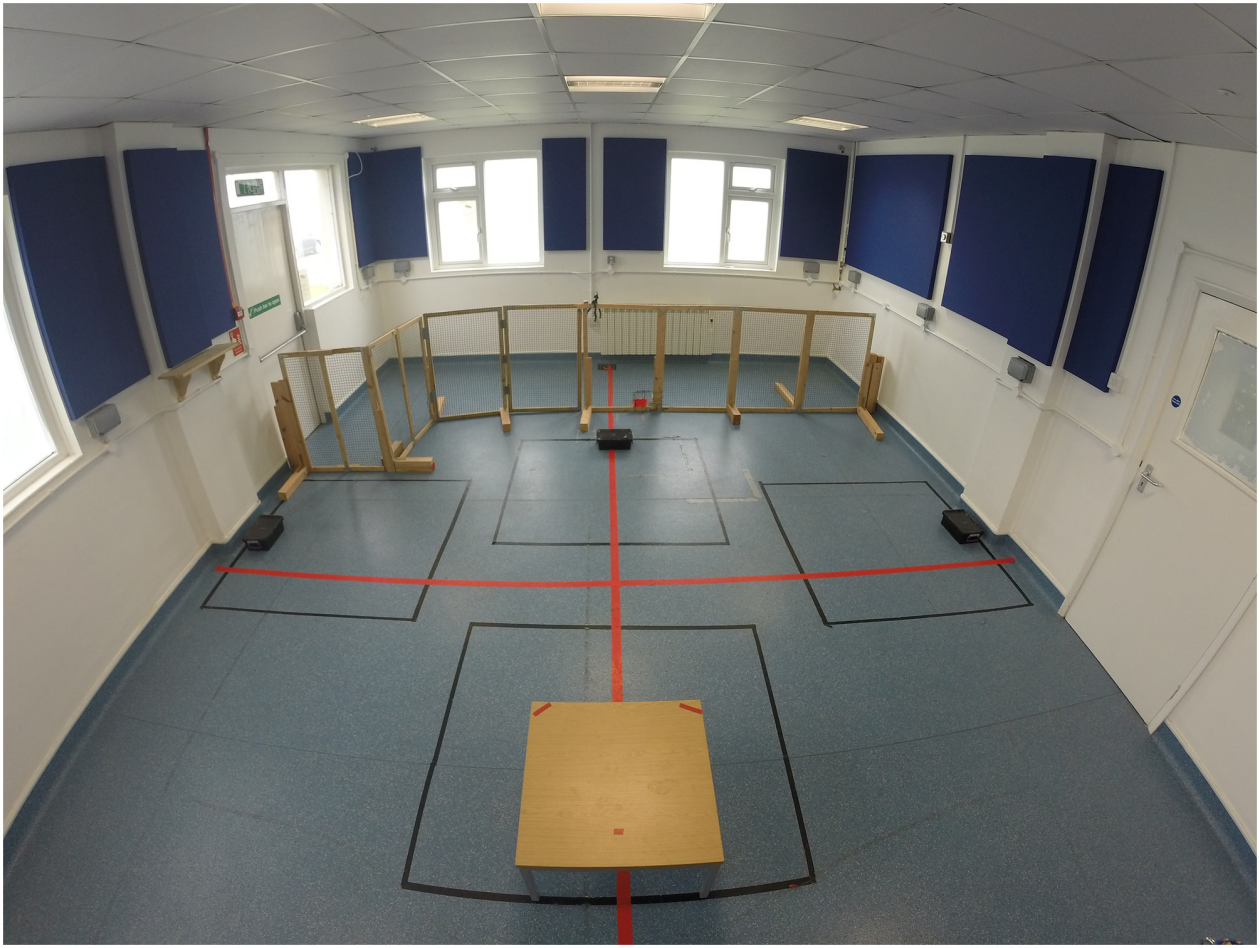
Testing room for study 2. A bench was positioned in the middle of the testing room. Three opaque containers (one on the left, one in front and one on the right of the bench) were positioned so that each of them was at the same distance from each other and from the bench. The two objects, relevant and distractor, were positioned on the bench before the dog entered the testing room.

Testing took place in one of the rooms (4.60 m x 4.20 m) of the Dog Cognition Centre Portsmouth (DOCS). Three opaque containers (19 cm x 10 cm) were placed on the floor: one in the left, one on the middle and the other in the right corner of the room. A bench for the experimenter to sit on was placed in the middle of the three containers and at a distance of 2.70 m to two of the containers and at a distance of 2.60m of the third (Fig 2). Two different objects were used as hidden targets: a notepad (*relevant object*) and a stapler (*distractor*).

Like in study 1, the procedure started with a warm-up phase. After the warm-up the dog was led out of the room by the helper and the experimenter. The dog and the experimenter re-entered the room and the experimenter sat down on the bench. The two objects, the notepad and the stapler, were lying on the bench. The experimenter ignored the stapler, and picked up the notepad to write her notes. In order to make sure the dog noticed her activity, the researcher continuously mumbled to herself while being busy writing. If the dog moved far away, the experimenter called for the dog’s attention to ensure he returned while never specifically indicating the notepad. After using the notepad for 30 sec (measured with a timer) the experimenter said something like “Oh, I need to leave, you wait here!” and left the room through door A while leaving the notepad on the bench.

After the experimenter left the room, the helper entered through the same door, went straight to the bench and picked up the notepad and the stapler. Then, making sure that the dog was watching, she hid one of the two objects depending on the condition while holding on to the other object. Dogs were randomly assigned to one of the two conditions:

*Relevant* condition: the helper hid the relevant object (the notepad) in one of the three boxes while catching the dog’s attention by talking to him while hiding the object.
*Distractor* condition: the helper hid the distractor (the stapler) in one of the three boxes while catching the dog’s attention by talking to him while hiding the object.

The helper always started the baiting of the containers by opening the containers to the left, then the middle one and finally the one on the right. While opening all containers she kept the dog’s attention by talking to the dog but did not pay more attention to any of the containers over the others. After the hiding was completed, the helper left the room through door B (Fig 2), taking with her the object she had not hidden, and leaving the dog in the testing room.

After the helper had left, the experimenter returned trough door A, and started the search following the exact same protocol as in study 1.

The study followed a mixed design. The between subjects variable was the group that dogs were allocated to. Within each group it was then varied whether the experimenter talked to the dog in a high-pitched voice while searching, “*vocal* trials”, or not, “*silent* trials” (within subject variable). Vocal and silent trials were presented blocked with half of the dogs in each group starting with vocal trials and the other half starting with silent trials. Dogs in each group (*relevant* and *distractor*) received three vocal and three silent trials summing up to six trials altogether. The location where the object was hidden was counterbalanced and semi-randomised following a double *Latin square* design so that during each block (silent and vocal) the object was hidden once in each container and the possible combinations were counterbalanced across the subjects. After the searching phase had elapsed the experimenter had to take a decision on which container to check. Again this was identical to the protocol used in Study 1. After making a choice the trial was over, the experimenter guided the dog out of the room and the helper entered the testing room to rearrange it for the following trial.

### Behavioural analysis

We recorded the frequency of gazes towards two distinctive locations in the room: (1) gazing at the experimenter, (2) gazing at the box where the target object was hidden (*target box*). As in study 1 gazes were subjected to a sequential analysis and *gaze alternations* were recorded.

As in study 1, the duration of looks toward the experimenter during the demonstration phase were also recorded.

Again, in order to assess inter-coder reliability a random selection of the video material (20%) was coded by a second observer, naïve to the purpose of the study and to the content of the hiding boxes. The correlation between the two coders was calculated using Spearman r. Inter-observer reliability was moderate for the frequency of gazes to the *target box* (r_s_ = .44, N = 58, p = .001) and the duration of gazes to the *target box* (r_s_ = .53, N = 58, p = .001). There was an excellent agreement on the frequency of gazes to the experimenter (r_s_ = .86, N = 58, p = .001), the duration of gazes to the experimenter (r_s_ = .90, N = 58, p = .001), and the duration of gazes during the demonstration (r_s_ = .88, N = 59, p = .001).

### Statistical analysis

Data were analysed using the statistical software R [56], with the packages *lme4* [56], *MuMIn* [57], and *lsmeans* [58]. A modelling approach (GLMM) was used for the analysis of the data using the same procedure applied to study 1. All results have been reported with standard errors.

A GLMM (null model) with log function was calculated with the count response variable “gaze alternations” (number of gaze alternations toward the target box), and the nested random intercept factors “dog”, “counterbalancing group” and “trial” (N = 288, number of subjects = 48). All the relevant fixed factors and interactions were included in the model (S1 Text for details). There were no significant main effects or interactions, therefore the null model was retained.

Another GLMM with logit function was calculated with the response variable “duration of gazes (s)”, weighted by the factor “duration of trials (s)” (null model). The random intercept factor “dog” (N = 48) was included in the null model. All the relevant fixed factors and interactions were included in the model (S1 Text for details). The model that yielded the lowest AIC comprised the fixed factors “direction” (experimenter, empty-boxes, target-box, other), “condition” (relevant, distractor), and “communication” (silent, vocal), with a 3 level interaction.

## Results

Nearly all dogs alternated their gazes between the experimenter and the *target box* (92% in the *relevant* 1group, 100% in the *distractor* group), with no significant difference between the two groups (Fisher’s exact test, *p* = .49).

The analysis of the frequencies indicated that the number of gaze alternations was not influenced by the condition (GLMM_Condition_, N = 48, χ^2^_1_ = 1.764, *p* = 0.184), or the communication (GLMM_Communication_, N = 48, χ^2^_1_ = 0.609, *p* = 0.435). Therefore any variation in the frequency of gaze alternations was due to individual differences.

**Fig 3:**
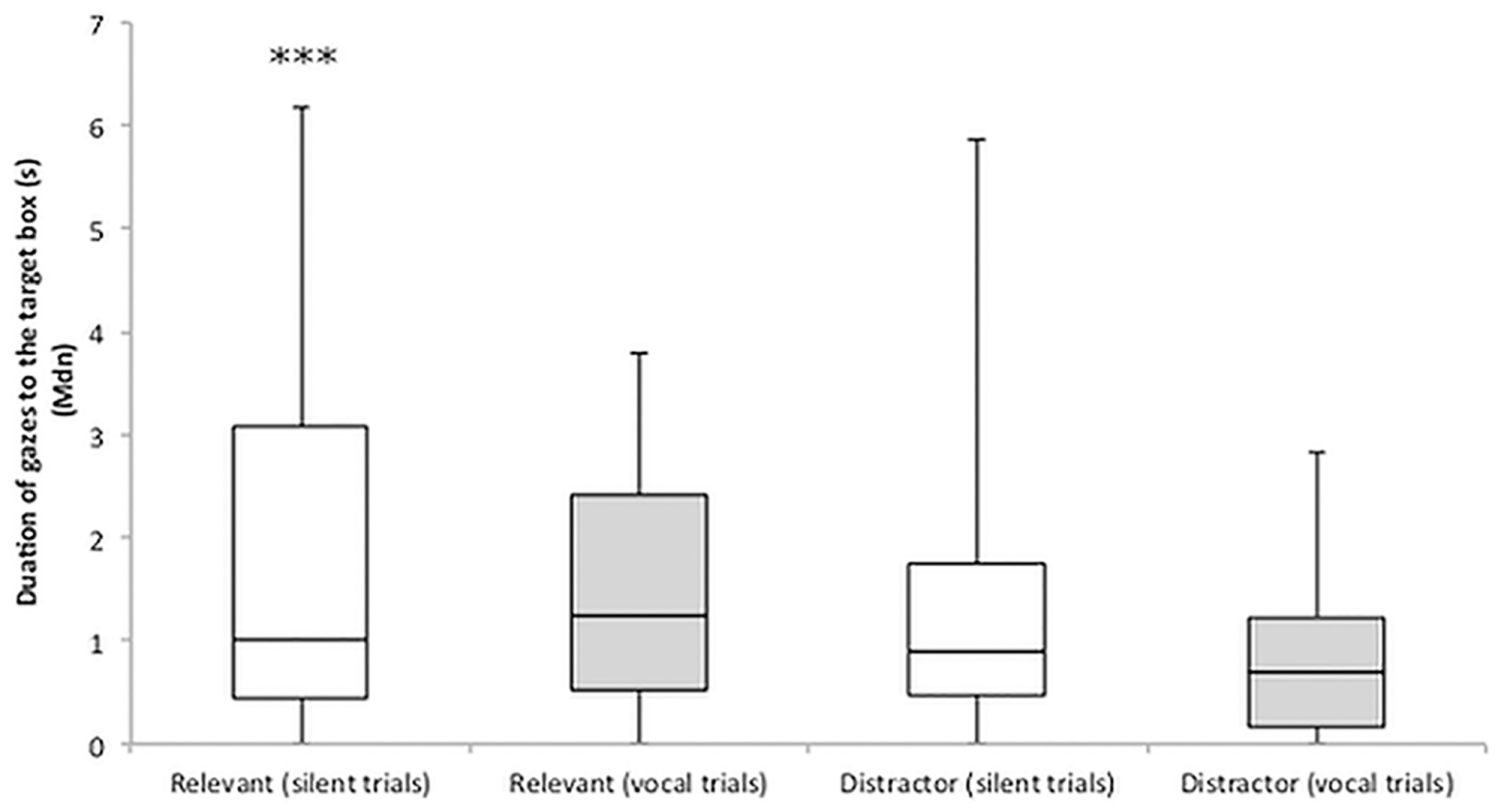
Effect of direction, condition, and communication on dogs’ gazes. Gaze persistency was more likely to increase when gazes were directed to the target, in the *relevant* group and in the vocal trials. A breakdown of the duration of gazes to the target, divided by condition and communication, is presented in the graph. The middle line in the box plots represent the median duration of gazes, the extremes of the boxes represent the lower and upper quartiles, and the error bars represent the minimum and maximum duration of gazes.

There was an effect, with a 3 level interaction, of the direction of the gaze, the content of the target box (condition), and the communication on the duration of dog gazes (GLMM_Direction*Condition*Communication_, *N* = 48, χ^2^_15_ = 1602, *p* = 0.001). The factor “attention” during the demonstration did not improve the model and was therefore not included (GLMM_Direction*Condition*Communication+Attention_, *N* = 48, χ^2^_1_ = 0, *p* = 0.995). Gaze duration was more likely to increase when dogs were gazing at the target (compared to an empty box), in the relevant group (compare to the distractor group), and in the vocal trials (compared to silent trials) (estimate _target*relevant*vocal_ ± SE = 0.336 ± 0.098, *p* = 0.001) (Fig 3).

## Discussion

The findings of this study showed that dogs seemed to differentiate between the objects that were hidden. Vocal trials and the presence of the relevant object led to more persistent, i.e. longer gazes directed to the target. This can possibly be an indicator that dogs differentiate the objects based on the humans interest in them and might mean that dogs communicative behaviour towards humans is underlined by a helpful motive, as it is similar to the infants’ informative pointing described by Liszkowski and colleagues [25,26]. A more parsimonious explanation is that the high pitch voice used by the experimenter had an arousing effect on dogs [67], thereby enhancing their communicative response. However, humans’ ostensive cues, in this case high-pitched voice, initiate and maintain communication in dogs [50,68]. Consequently, another possibility is that the experimenter’s voice helped in establishing a communicative context or helped the dogs understanding the humans’ need for information. Future research could further investigate how different types of ostensive cues affect dogs’ communication. Recent results showed that temporal contiguity between human ostensive cues and referential signals (pointing) is necessary for dogs to understand the gesture. The manipulation of the temporal order in which ostensive cues and pointing were presented to the dog, in fact, allowed for the confirmation of the importance of ostensive signals preceding referential cues in communication-based knowledge acquisition processes in dogs [68]. Also eye contact with the owner increases dogs’ attention getting behaviours [69]. The systematic manipulation of different ostensive cues (e.g. high pitch voice, eye contact), in association with their temporal manipulation (before and after searching behaviour) [70] may aid the understanding the role of high pitch voice upon dogs’ behaviour in a cooperative-communicative context. Applying such an approach to a range of communicative and non-communicative contexts could possibly allow teasing apart the overall arousing effect of some ostensive cues (i.e. high pitch sounds) from the more context specific effects on dogs’ communication.

## General Discussion

The results of study 1 show that dogs did not indicate preferentially the object needed by the experimenter. They rather indicated objects that they had an interest in (i.e. the toy or novel objects). However, the dogs’ indications were more persistent when directed to the relevant object, and increased with the attention during the demonstration. These results are confirmed by those of study 2 where, in the absence of a personal interest, dogs’ indications towards an object relevant for the human were more persistent when compared to indications towards a distractor if the experimenter verbally addressed the dog. In the light of these results, there seems to be some evidence that dogs could be able to distinguish between objects based on a human’s need for them. Interestingly, in both studies dogs used gaze alternation with similar frequency regardless of the relevance of the object, therefore indicating that objects’ relevance may not affect the motivation of dogs to establish joint attention when communicating to humans.

The use of contingencies between the events observed by the dogs could be a more parsimonious mechanism that may as well possibly explain these results. Stimulus enhancement, caused by witnessing the experimenter interacting with the relevant object, could have directed the behaviour of the dogs. Such a possibility would imply that the dogs did not understand the relevance of the object to the experimenter. Although the helper manipulated both objects in all conditions in an attempt to control for this, the possibility cannot be completely excluded. However, the level of flexibility with which dogs use their *showing behaviour* [19,23,24,71] makes this mechanism less likely to be the sole explanation for their communicative behaviour.

Another possible explanation for our results is that dogs’ communication may be underlined by *informative* motives. Gaze alternations show dogs’ intention to form joint attention with the experimenter [19], while the persistent gazes towards the relevant object may have been used to direct the experimenter’s attention [39]. Such behaviour is consistent with the description of *informative* pointing provided by Liszkowski and colleague, where the pointer provides the information by directing the recipient’s attention towards a target because of the recipient’s relation to the target itself, rather than a personal interest [25]. For this to be possible dogs need to possess a number of skills. In order to understand the human’s need for information, dogs need to recognise humans as intentional agents [49], as well as have the motivation to use communication helpfully [25]. Dogs perceive the communicative intent in the human pointing, as demonstrated by their ability to distinguish an intentional communicative pointing from similar, non-communicative movements in the same direction [63]. Moreover, Marshall-Pescini and colleagues, using a habituation-dishabituation paradigm, were able to show that dogs appear to perceive human actions as goal-directed [72]. Finally, dogs have been selected during domestication for being particularly skilful in interacting with humans in social and communicative situations [12,18,73]. There are indications that they have helpful motives when interacting with humans in general, such as during instrumental helping [74], cooperative problem solving [75], and complex cooperative interactions [76,77]. Additionally, dogs also have the general motivation to act cooperatively in response to humans’ requests [49].

Another parsimonious explanation for our results could possibly be that dogs were indicating the hidden object to comply with a human request, as previously suggested by Kaminski and colleagues [49]. It has been hypothesised that dogs interpret human referential behaviour as being about something but cannot make the connection to the specific object that is being referred to [78]. It is possible that dogs interpret human search and ostensive cues as directives, e.g. a request to fetch or to find a hidden object [49,51].

Moore and Gomez propose that, in ape and infant pointing, imperative and declarative gestures could possibly share the common cognitive complexity of understanding behaviours as connected to targets through joint attention [38,39,79]. The dogs in our study established joint attention in both conditions. Therefore this interpretation could be valid for dogs as well. This could imply that dogs possibly indicated the hidden object because they interpreted it as the target of the experimenter’s search, especially in the case of the *distractor* group in study 2, when the *relevant* object was not in the room and there were no other objects attracting the attention of the dogs. Such a mechanism is similar to that described by Csibra and Gergely, and according to the authors it does not require the understanding of others’ mental states and is possible in non-human animals [40,41,43]. Nevertheless, the possibility of *informative* communication is not excluded. Specifically, the fact that dogs’ *showing behaviours* were more persistent in the *relevant* condition, demonstrates that at least in the *relevant* condition, dogs took into account the relevance of the objects to the experimenter when communicating. This could not be explained by a more parsimonious mechanism, such as social enhancement. On the contrary, interpretations such those of Moore and Gomez do not require the understanding of humans’ state of knowledge or the intent to influence the mental state of others. It would suffice for dogs to recognise the communicative context, e.g. through the human ostensive cues, and to identify the relevant object as the target of the human’s search in order to indicate a target relevant for the receiver [38,39].

In conclusion, while the current results could not demonstrate the presence of an informative intent in dogs’ communication, they do not fully exclude this possibility, which needs further investigation. Specifically, this study provides some evidence that dogs may be able to recognise the relevance of an object for a human partner based on the context in which it was used. Further research should attempt to tease apart the elements driving dogs’ understanding of objects’ relevance. Coincidentally, the results add to the existing body of evidence indicating some level of a helpful motivation in dogs’ communication, demonstrating that such helpful drive is easily masked by preponderant selfish interests. When more preferred objects were not present in the room (study 2), dogs indicated targets that they had no interest in, without receiving any explicit reward. It may therefore be necessary to account for competing interests when investigating helpful motives in dogs.

## Supporting Information

S1 Dataset Raw data for study 1 and study 2. (PDF)

S1 Text Statistical analysis: model fitting additional information for study 1 and study 2. (PDF)
