# Supplementary material for: Do Dogs Provide Information Helpfully?
Source: PLoS One. 2016 Aug 10;11(8):e0159797. doi: 10.1371/journal.pone.0159797 (PMC4980001; doi:10.1371/journal.pone.0159797)
Supplement: S1 Dataset — (PDF) [file pone.0159797.s001.pdf]

Data set for study 1

Dog	Trial	Gender	Age	Breed	Condition	Group	ToySide	Duration Gaze Toy	Duration Gaze Target	Frequency Gaze Alternation Toy	Frequency Gaze Alternation Target	Attention Demo	Indication Target Before Utterance	Indication Target After Utterance
Ashka	1	Female	2	Cross	empty	5	left	21.3	5.6	6	0	13.2	0	1
Ashka	2	Female	2	Cross	empty	5	right	5.1	0	3	0	13.6	1	1
Ashka	3	Female	2	Cross	tool	5	right	2.5	1.4	4	2	11.9	0	0
Ashka	4	Female	2	Cross	tool	5	left	0	0	0	0	6.2	1	0
Ashka	5	Female	2	Cross	random	5	left	5.1	1.4	1	1	31.9	1	1
Ashka	6	Female	2	Cross	random	5	right	0	0.6	0	0	6.8	1	1
Bailey	1	Female	4	Cross	tool	2	left	0.6	0	0	0	6.8	0	0
Bailey	2	Female	4	Cross	tool	2	right	0	0.5	0	1	14.1	1	0
Bailey	3	Female	4	Cross	empty	2	right	0.6	0	2	0	4.9	1	0
Bailey	4	Female	4	Cross	empty	2	left	12.7	0.8	4	0	10.3	0	0
Bailey	5	Female	4	Cross	random	2	right	2.1	2.1	2	1	52.4	0	0
Bailey	6	Female	4	Cross	random	2	left	19.2	0	0	0	11.7	0	0
Boomer	1	Female	3.5	Gundog	random	3	right	0	0.7	0	0	8.4	0	0
Boomer	2	Female	3.5	Gundog	random	3	left	1.9	3.1	3	1	20.3	1	1
Boomer	3	Female	3.5	Gundog	tool	3	left	4	0.8	0	0	5.9	0	0
Boomer	4	Female	3.5	Gundog	tool	3	right	0	0	0	0	10.2	0	0
Boomer	5	Female	3.5	Gundog	empty	3	left	0	0	0	0	22.2	1	0
Boomer	6	Female	3.5	Gundog	empty	3	right	1.2	0	1	0	15.7	0	1
Charlie	1	Male	2.5	Cross	empty	6	right	1.4	1	2	2	16.4	1	1
Charlie	2	Male	2.5	Cross	empty	6	left	5.6	2.1	2	3	51.2	0	1
Charlie	3	Male	2.5	Cross	random	6	right	5.8	2.6	0	0	13.3	0	1
Charlie	4	Male	2.5	Cross	random	6	left	2.5	1	2	1	19.3	0	1
Charlie	5	Male	2.5	Cross	tool	6	left	1	1.1	1	1	22.5	0	1
Charlie	6	Male	2.5	Cross	tool	6	right	0.6	1	1	2	8.7	0	1
Crusoe	1	Male	4	Working	tool	1	left	3.1	4.2	2	2	46.8	0	0
Crusoe	2	Male	4	Working	tool	1	right	3.8	0.7	1	2	55.3	1	1
Crusoe	3	Male	4	Working	random	1	right	0.5	0.6	0	0	58.5	1	1
Crusoe	4	Male	4	Working	random	1	left	1	0.5	1	1	0.0	1	0
Crusoe	5	Male	4	Working	empty	1	right	0	0	0	0	54.7	0	1
Crusoe	6	Male	4	Working	empty	1	left	0	0	0	0	43.7	0	0
Dakota	1	Female	2	Cross	tool	1	right	2.5	3.5	2	1	11.4	0	0
Dakota	2	Female	2	Cross	tool	1	left	7.2	0.5	3	0	5.0	0	0
Dakota	3	Female	2	Cross	random	1	left	4	1.2	0	2	19.2	1	1
Dakota	4	Female	2	Cross	random	1	right	0.7	0	0	0	14.5	0	0
Dakota	5	Female	2	Cross	empty	1	left	0	0	0	0	19.6	0	0
Dakota	6	Female	2	Cross	empty	1	right	1.4	2.5	2	1	1.0	1	1
Harry	1	Male	3.5	Cross	empty	6	left	4.4	9.4	1	6	17.9	0	1
Harry	2	Male	3.5	Cross	empty	6	right	7	0	9	0	4.7	1	0
Harry	3	Male	3.5	Cross	random	6	left	2.3	4.7	2	6	7.6	0	1
Harry	4	Male	3.5	Cross	random	6	right	4	2.3	6	2	20.6	0	1
Harry	5	Male	3.5	Cross	tool	6	left	6.8	2.3	5	3	7.2	0	0
Harry	6	Male	3.5	Cross	tool	6	right	0.6	1	1	1	9.3	0	0
Hudson	1	Male	4	Terrier	random	4	left	0.9	4.1	1	5	29.8	0	1
Hudson	2	Male	4	Terrier	random	4	right	1.4	1.6	2	1	21.2	1	0
Hudson	3	Male	4	Terrier	empty	4	left	1.7	0.5	2	2	9.7	1	1
Hudson	4	Male	4	Terrier	empty	4	right	2.4	0.6	3	1	20.0	0	0
Hudson	5	Male	4	Terrier	tool	4	left	1.4	1.5	1	3	2.5	0	1
Hudson	6	Male	4	Terrier	tool	4	right	12.4	0.8	5	1	13.2	0	0
Iggy	1	Male	5	Gundog	random	3	right	2.8	0	2	0	15.8	0	0
Iggy	2	Male	5	Gundog	random	3	left	1.1	2	2	1	16.1	0	1
Iggy	3	Male	5	Gundog	tool	3	left	0	0.5	0	2	14.7	0	0
Iggy	4	Male	5	Gundog	tool	3	right	0.9	0.7	1	0	21.2	0	1
Iggy	5	Male	5	Gundog	empty	3	right	0	0	0	0	27.1	1	0
Iggy	6	Male	5	Gundog	empty	3	left	0.6	0	2	0	9.9	0	0
Jeff	1	Male	7.5	Terrier	tool	2	left	0.5	3.2	0	2	26.5	0	0
Jeff	2	Male	7.5	Terrier	tool	2	right	2.4	4.3	4	0	34.1	0	0
Jeff	3	Male	7.5	Terrier	empty	2	left	0	2.4	0	3	8.8	1	1
Jeff	4	Male	7.5	Terrier	empty	2	right	0.9	0.7	1	1	39.4	0	0
Jeff	5	Male	7.5	Terrier	random	2	right	0	0	0	0	29.9	1	1
Jeff	6	Male	7.5	Terrier	random	2	left	0.5	3.8	0	1	17.9	1	1
Koko	1	Female	3.5	Hound	empty	5	right	0.5	1.7	1	0	20.0	1	1
Koko	2	Female	3.5	Hound	empty	5	left	1.6	4.2	0	1	16.7	1	1
Koko	3	Female	3.5	Hound	tool	5	left	6.2	1.3	3	0	14.9	0	0
Koko	4	Female	3.5	Hound	tool	5	right	0	2.7	0	1	10.4	0	0
Koko	5	Female	3.5	Hound	random	5	left	0.5	1.5	0	0	16.3	1	1
Koko	6	Female	3.5	Hound	random	5	right	1.9	2.7	2	2	5.0	1	0
Lanson	1	Male	2	Gundog	random	4	right	0	0	0	0	6.9	1	1
Lanson	2	Male	2	Gundog	random	4	left	0	0	0	0	12.4	0	0
Lanson	3	Male	2	Gundog	empty	4	left	0	0	0	0	18.3	1	1
Lanson	4	Male	2	Gundog	empty	4	right	0.9	0.8	0	0	18.6	0	0
Lanson	5	Male	2	Gundog	tool	4	right	0	0	0	0	17.2	0	0
Lanson	6	Male	2	Gundog	tool	4	left	0	0	0	0	32.6	1	1
Maddie	1	Female	3.5	Utility	tool	2	left	4.9	0	0	0	4.3	0	1
Maddie	2	Female	3.5	Utility	tool	2	right	0.6	1.9	0	0	18.2	0	0
Maddie	3	Female	3.5	Utility	empty	2	left	2	0	0	0	11.1	0	0
Maddie	4	Female	3.5	Utility	empty	2	right	1.7	5.2	0	0	4.8	1	0
Maddie	5	Female	3.5	Utility	random	2	left	3.2	0.6	0	0	20.8	1	1
Maddie	6	Female	3.5	Utility	random	2	right	0	2.9	0	0	11.7	1	1
MaxM	1	Male	8	Cross	tool	1	right	16.5	0.9	3	1	16.4	0	0
MaxM	2	Male	8	Cross	tool	1	left	10.8	1.3	3	2	13.4	0	1
MaxM	3	Male	8	Cross	random	1	right	6.1	2.7	0	1	28.1	1	0
MaxM	4	Male	8	Cross	random	1	left	4.5	5.3	1	5	14.9	1	1
MaxM	5	Male	8	Cross	empty	1	left	3.5	1	1	3	25.1	1	1
MaxM	6	Male	8	Cross	empty	1	right	5.8	7.9	0	3	30.6	1	1
Millie	1	Female	1.5	Gundog	tool	1	left	0	0.9	0	0	4.7	0	1
Millie	2	Female	1.5	Gundog	tool	1	right	2.6	0	2	0	18.8	1	1
Millie	3	Female	1.5	Gundog	random	1	right	0	0	0	0	13.9	0	1
Millie	4	Female	1.5	Gundog	random	1	left	0.5	0	0	0	17.1	0	1
Millie	5	Female	1.5	Gundog	empty	1	left	1.1	0	1	0	15.0	1	1
Millie	6	Female	1.5	Gundog	empty	1	right	0	0	0	0	18.6	1	0
Moet	1	Male	4	Gundog	random	4	left	0.5	1.7	0	0	10.0	0	1
Moet	2	Male	4	Gundog	random	4	right	5.7	0.7	1	0	14.8	0	0
Moet	3	Male	4	Gundog	empty	4	right	0	0	0	0	11.6	1	0
Moet	4	Male	4	Gundog	empty	4	left	0	0	0	0	10.7	0	0
Moet	5	Male	4	Gundog	tool	4	right	0	0	0	0	0.0	1	1
Moet	6	Male	4	Gundog	tool	4	left	0	0	0	0	10.9	0	0
Moses	1	Female	6	Cross	random	3	right	0.6	0	1	0	27.1	1	0
Moses	2	Female	6	Cross	random	3	left	0	0	0	0	2.8	0	0
Moses	3	Female	6	Cross	tool	3	left	0	0	0	0	12.4	0	0
Moses	4	Female	6	Cross	tool	3	right	3.1	0.5	2	0	0.0	0	0
Moses	5	Female	6	Cross	empty	3	left	1.5	0.8	0	0	18.1	0	1
Moses	6	Female	6	Cross	empty	3	right	0	0	0	0	7.3	0	0
PoppyP	1	Female	3	Cross	random	3	right	1	0	4	0	8.4	0	0
PoppyP	2	Female	3	Cross	random	3	left	1.5	0	2	0	19.9	0	1
PoppyP	3	Female	3	Cross	tool	3	left	1	1.2	1	1	7.0	0	1
PoppyP	4	Female	3	Cross	tool	3	right	0.5	0	1	0	9.6	0	0
PoppyP	5	Female	3	Cross	empty	3	left	7.6	11.7	0	0	13.3	1	1
PoppyP	6	Female	3	Cross	empty	3	right	1.1	1.4	1	1	3.1	0	0
Rigsby	1	Male	5	Cross	empty	6	left	0	0	0	0	5.1	1	1
Rigsby	2	Male	5	Cross	empty	6	right	0	0.5	0	0	6.3	0	0
Rigsby	3	Male	5	Cross	random	6	left	0	2.5	0	1	0.0	1	1
Rigsby	4	Male	5	Cross	random	6	right	6.7	0	0	0	37.5	1	1
Rigsby	5	Male	5	Cross	tool	6	left	0	0	0	0	3.8	0	0
Rigsby	6	Male	5	Cross	tool	6	right	0	0	0	0	7.7	1	1
Rumsey	1	Male	2.5	Cross	empty	5	left	6.3	1.5	3	1	5.4	1	0
Rumsey	2	Male	2.5	Cross	empty	5	right	4.2	1	0	1	4.1	1	0
Rumsey	3	Male	2.5	Cross	tool	5	right	5	0.7	2	0	14.5	0	1
Rumsey	4	Male	2.5	Cross	tool	5	left	0	0	0	0	57.8	1	1
Rumsey	5	Male	2.5	Cross	random	5	left	0	4.7	0	0	11.2	1	1
Rumsey	6	Male	2.5	Cross	random	5	right	0	20.3	0	0	6.2	0	0
Sailor	1	Male	1.5	Gundog	tool	2	right	3.9	0	2	0	33.9	1	1
Sailor	2	Male	1.5	Gundog	tool	2	left	3.5	2	2	0	2.8	0	1
Sailor	3	Male	1.5	Gundog	empty	2	right	9.7	9.6	0	1	14.3	0	0
Sailor	4	Male	1.5	Gundog	empty	2	left	0.5	4.1	1	0	0.0	1	1
Sailor	5	Male	1.5	Gundog	random	2	left	2.8	3.8	0	1	4.2	0	1
Sailor	6	Male	1.5	Gundog	random	2	right	0.5	3.3	1	1	1.0	0	0
Storm	1	Female	3	Cross	empty	5	right	0.5	0	2	0	9.4	1	0
Storm	2	Female	3	Cross	empty	5	left	0.8	3.6	1	3	39.2	1	1
Storm	3	Female	3	Cross	tool	5	right	2.7	0	2	0	14.8	1	0
Storm	4	Female	3	Cross	tool	5	left	0.5	0	0	0	29.2	0	1
Storm	5	Female	3	Cross	random	5	right	0.7	1.9	1	1	16.7	1	0
Storm	6	Female	3	Cross	random	5	left	1.9	0.7	1	2	20.5	1	0
Winston	1	Male	4	Cross	random	4	left	0.5	0	1	0	11.3	1	1
Winston	2	Male	4	Cross	random	4	right	0.5	1.6	1	0	0.0	1	0
Winston	3	Male	4	Cross	empty	4	left	0	0	0	0	9.8	0	1
Winston	4	Male	4	Cross	empty	4	right	0	0	0	0	2.7	0	0
Winston	5	Male	4	Cross	tool	4	left	0.5	0	1	0	16.5	0	1
Winston	6	Male	4	Cross	tool	4	right	0	0	0	0	6.3	1	1
Woody	1	Male	6	Pastoral	empty	6	left	0.7	0	1	0	45.2	1	1
Woody	2	Male	6	Pastoral	empty	6	right	2.5	0	2	0	11.4	0	0
Woody	3	Male	6	Pastoral	random	6	right	0.9	2.6	0	2	22.1	0	0
Woody	4	Male	6	Pastoral	random	6	left	0	0	0	0	23.0	0	0
Woody	5	Male	6	Pastoral	tool	6	right	1.8	0	2	0	38.3	1	1
Woody	6	Male	6	Pastoral	tool	6	left	0	0	0	0	10.5	1	1

Dataset for study 2

Dog	Gender	Breed	Age	Trial	Condition	Communication	Group	Frequency Gaze Alternation Target	Duration Gaze Target	Frequency Gaze Alternation Empty Box	Duration Gaze Box	Attention Demo
Alpha	Male	Cross	8	1	Irrelevant	Vocal	Irrelevant, Vocal-Silent	1	2	0	0	33.87
Alpha	Male	Cross	8	2	Irrelevant	Vocal	Irrelevant, Vocal-Silent	1	0.5	0.5	0.85	11.08
Alpha	Male	Cross	8	3	Irrelevant	Vocal	Irrelevant, Vocal-Silent	2	0.5	1	0.7	20.87
Alpha	Male	Cross	8	4	Irrelevant	Silent	Irrelevant, Vocal-Silent	1	1.2	0.5	0.3	19.64
Alpha	Male	Cross	8	5	Irrelevant	Silent	Irrelevant, Vocal-Silent	1	0.6	0.5	0.3	19.11
Alpha	Male	Cross	8	6	Irrelevant	Silent	Irrelevant, Vocal-Silent	1	0.6	1	0.95	17.54
Arffer	Male	Cross	6	1	Irrelevant	Silent	Irrelevant, Silent-Vocal	1	7.5	0	0.7	29.62
Arffer	Male	Cross	6	2	Irrelevant	Silent	Irrelevant, Silent-Vocal	0	8.7	0	0	46.65
Arffer	Male	Cross	6	3	Irrelevant	Silent	Irrelevant, Silent-Vocal	1	0.8	0.5	4.4	30.89
Arffer	Male	Cross	6	4	Irrelevant	Vocal	Irrelevant, Silent-Vocal	0	2.1	0	0.65	12.83
Arffer	Male	Cross	6	5	Irrelevant	Vocal	Irrelevant, Silent-Vocal	1	2.5	1	3.6	14.06
Arffer	Male	Cross	6	6	Irrelevant	Vocal	Irrelevant, Silent-Vocal	0	0	0.5	7.2	11.25
Bear	Male	Cross	1	1	Irrelevant	Silent	Irrelevant, Silent-Vocal	0	0.9	0	0.9	24.25
Bear	Male	Cross	1	2	Irrelevant	Silent	Irrelevant, Silent-Vocal	0	0	1.5	2.6	39.62
Bear	Male	Cross	1	3	Irrelevant	Silent	Irrelevant, Silent-Vocal	1	3.1	0.5	1.65	34.89
Bear	Male	Cross	1	4	Irrelevant	Vocal	Irrelevant, Silent-Vocal	1	2.8	0	0	13.35
Bear	Male	Cross	1	5	Irrelevant	Vocal	Irrelevant, Silent-Vocal	0	0	1.5	0.65	12.10
Bear	Male	Cross	1	6	Irrelevant	Vocal	Irrelevant, Silent-Vocal	0	0.8	0.5	1.5	31.96
Bella	Female	Gundog	2	1	Relevant	Vocal	Relevant, Vocal-Silent	0	0	2	3.3	43.17
Bella	Female	Gundog	2	2	Relevant	Vocal	Relevant, Vocal-Silent	3	4	1.5	1.3	8.12
Bella	Female	Gundog	2	3	Relevant	Vocal	Relevant, Vocal-Silent	2	3.4	0	0	18.77
Bella	Female	Gundog	2	4	Relevant	Silent	Relevant, Vocal-Silent	0	0	1	0.4	16.35
Bella	Female	Gundog	2	5	Relevant	Silent	Relevant, Vocal-Silent	0	0	1.5	0.55	9.26
Bella	Female	Gundog	2	6	Relevant	Silent	Relevant, Vocal-Silent	0	0	0.5	0.4	11.88

Ben	Male	Cross	1.5	1	Relevant	Vocal	Relevant, Vocal-Silent	0	0	1	0.25	23.78
Ben	Male	Cross	1.5	2	Relevant	Vocal	Relevant, Vocal-Silent	1	1	0	0	19.57
Ben	Male	Cross	1.5	3	Relevant	Vocal	Relevant, Vocal-Silent	0	0.8	0	0	32.69
Ben	Male	Cross	1.5	4	Relevant	Silent	Relevant, Vocal-Silent	1	0.6	1	3.1	38.54
Ben	Male	Cross	1.5	5	Relevant	Silent	Relevant, Vocal-Silent	1	0.9	0	0	19.54
Ben	Male	Cross	1.5	6	Relevant	Silent	Relevant, Vocal-Silent	1	0.5	0.5	0.45	10.95
Blue	Male	Gundog	9.5	1	Irrelevant	Vocal	Irrelevant, Vocal-Silent	0	6.2	0	0	17.05
Blue	Male	Gundog	9.5	2	Irrelevant	Vocal	Irrelevant, Vocal-Silent	0	0.5	0	0.65	11.67
Blue	Male	Gundog	9.5	3	Irrelevant	Vocal	Irrelevant, Vocal-Silent	2	1.2	0	1.55	60.38
Blue	Male	Gundog	9.5	4	Irrelevant	Silent	Irrelevant, Vocal-Silent	0	0	1	0.55	24.28
Blue	Male	Gundog	9.5	5	Irrelevant	Silent	Irrelevant, Vocal-Silent	0	0	1	0.7	35.35
Blue	Male	Gundog	9.5	6	Irrelevant	Silent	Irrelevant, Vocal-Silent	0	0	0	0	32.23
Bob	Male	Cross	3	1	Relevant	Silent	Relevant, Silent-Vocal	1	2.1	1	0.25	38.13
Bob	Male	Cross	3	2	Relevant	Silent	Relevant, Silent-Vocal	0	0	0	1.8	52.37
Bob	Male	Cross	3	3	Relevant	Silent	Relevant, Silent-Vocal	1	0.5	1	1.8	18.86
Bob	Male	Cross	3	4	Relevant	Vocal	Relevant, Silent-Vocal	2	0.5	0	0	23.96
Bob	Male	Cross	3	5	Relevant	Vocal	Relevant, Silent-Vocal	2	3.1	0	0	7.79
Bob	Male	Cross	3	6	Relevant	Vocal	Relevant, Silent-Vocal	0	0	0	0	62.21
Bollinger	Male	Cross	7.5	1	Irrelevant	Silent	Irrelevant, Silent-Vocal	2	0.7	0	0.25	17.11
Bollinger	Male	Cross	7.5	2	Irrelevant	Silent	Irrelevant, Silent-Vocal	0	0	0	0	8.51
Bollinger	Male	Cross	7.5	3	Irrelevant	Silent	Irrelevant, Silent-Vocal	0	0	1	0.65	32.62
Bollinger	Male	Cross	7.5	4	Irrelevant	Vocal	Irrelevant, Silent-Vocal	0	0	0	0	25.41
Bollinger	Male	Cross	7.5	5	Irrelevant	Vocal	Irrelevant, Silent-Vocal	0	0	0	0.25	26.86
Bollinger	Male	Cross	7.5	6	Irrelevant	Vocal	Irrelevant, Silent-Vocal	0	0	0	0	10.74
Bonnie	Male	Cross	2	1	Irrelevant	Silent	Irrelevant, Silent-Vocal	0	0	0.5	0.6	11.86
Bonnie	Male	Cross	2	2	Irrelevant	Silent	Irrelevant, Silent-Vocal	0	3.8	0	0	8.19
Bonnie	Male	Cross	2	3	Irrelevant	Silent	Irrelevant, Silent-Vocal	0	0	0	0.45	13.38
Bonnie	Male	Cross	2	4	Irrelevant	Vocal	Irrelevant, Silent-Vocal	0	0.6	1	0.25	25.73
Bonnie	Male	Cross	2	5	Irrelevant	Vocal	Irrelevant, Silent-Vocal	0	0	0	0	30.63
Bonnie	Male	Cross	2	6	Irrelevant	Vocal	Irrelevant, Silent-Vocal	0	0	0	0.4	4.93
Brian	Male	Gundog	5	1	Relevant	Silent	Relevant, Silent-Vocal	0	0	0	0.45	39.22
Brian	Male	Gundog	5	2	Relevant	Silent	Relevant, Silent-Vocal	0	0	0	1.4	14.74
Brian	Male	Gundog	5	3	Relevant	Silent	Relevant, Silent-Vocal	0	3.1	0	3.9	13.62
Brian	Male	Gundog	5	4	Relevant	Vocal	Relevant, Silent-Vocal	0	5.7	0	0.5	16.61
Brian	Male	Gundog	5	5	Relevant	Vocal	Relevant, Silent-Vocal	3	1.5	0	0.75	14.41
Brian	Male	Gundog	5	6	Relevant	Vocal	Relevant, Silent-Vocal	2	1.9	0	0	15.14
Brocken	Male	Gundog	3	1	Irrelevant	Vocal	Irrelevant, Vocal-Silent	0	0	0	0	22.15
Brocken	Male	Gundog	3	2	Irrelevant	Vocal	Irrelevant, Vocal-Silent	0	0	0	0	16.34
Brocken	Male	Gundog	3	3	Irrelevant	Vocal	Irrelevant, Vocal-Silent	1	0.5	0.5	0.5	23.38
Brocken	Male	Gundog	3	4	Irrelevant	Silent	Irrelevant, Vocal-Silent	0	0	0	0	19.38
Brocken	Male	Gundog	3	5	Irrelevant	Silent	Irrelevant, Vocal-Silent	0	0	1	0.25	43.71
Brocken	Male	Gundog	3	6	Irrelevant	Silent	Irrelevant, Vocal-Silent	0	0	2.5	1.05	1.60
Buzz	Male	Cross	2	1	Irrelevant	Vocal	Irrelevant, Vocal-Silent	0	0	0	0	11.15
Buzz	Male	Cross	2	2	Irrelevant	Vocal	Irrelevant, Vocal-Silent	0	0	0	0.65	26.56
Buzz	Male	Cross	2	3	Irrelevant	Vocal	Irrelevant, Vocal-Silent	1	0	0	0.5	19.24
Buzz	Male	Cross	2	4	Irrelevant	Silent	Irrelevant, Vocal-Silent	3	1	0	0	39.70
Buzz	Male	Cross	2	5	Irrelevant	Silent	Irrelevant, Vocal-Silent	1	2.7	0	1.2	13.49
Buzz	Male	Cross	2	6	Irrelevant	Silent	Irrelevant, Vocal-Silent	0	1.6	0	0.35	30.16
BuzzG	Male	Cross	3.5	1	Irrelevant	Vocal	Irrelevant, Vocal-Silent	0	0	0	4.45	28.21
BuzzG	Male	Cross	3.5	2	Irrelevant	Vocal	Irrelevant, Vocal-Silent	0	0.5	0.5	5.5	2.87
BuzzG	Male	Cross	3.5	3	Irrelevant	Vocal	Irrelevant, Vocal-Silent	0	2.8	0	0	17.70
BuzzG	Male	Cross	3.5	4	Irrelevant	Silent	Irrelevant, Vocal-Silent	0	0	1	1	15.81
BuzzG	Male	Cross	3.5	5	Irrelevant	Silent	Irrelevant, Vocal-Silent	0	0	0	0	35.53
BuzzG	Male	Cross	3.5	6	Irrelevant	Silent	Irrelevant, Vocal-Silent	2	1.5	0	0	11.73
Cassidy	Male	Cross	2	1	Relevant	Vocal	Relevant, Vocal-Silent	0	0	1	0.6	50.97
Cassidy	Male	Cross	2	2	Relevant	Vocal	Relevant, Vocal-Silent	1	0.5	0	0	38.91
Cassidy	Male	Cross	2	3	Relevant	Vocal	Relevant, Vocal-Silent	0	0	1	0.3	17.92
Cassidy	Male	Cross	2	4	Relevant	Silent	Relevant, Vocal-Silent	1	0.5	0.5	0.25	36.09
Cassidy	Male	Cross	2	5	Relevant	Silent	Relevant, Vocal-Silent	0	0	0	0.3	18.15
Cassidy	Male	Cross	2	6	Relevant	Silent	Relevant, Vocal-Silent	0	0	0	0	10.77
Daisy	Female	Cross	2.5	1	Relevant	Silent	Relevant, Silent-Vocal	1	0	0.5	0.35	22.03
Daisy	Female	Cross	2.5	2	Relevant	Silent	Relevant, Silent-Vocal	0	0.5	0.5	0.65	42.63
Daisy	Female	Cross	2.5	3	Relevant	Silent	Relevant, Silent-Vocal	1	0.6	1.5	1.65	33.86
Daisy	Female	Cross	2.5	4	Relevant	Vocal	Relevant, Silent-Vocal	0	0	0	0	38.01
Daisy	Female	Cross	2.5	5	Relevant	Vocal	Relevant, Silent-Vocal	0	0	0	0	39.09
Daisy	Female	Cross	2.5	6	Relevant	Vocal	Relevant, Silent-Vocal	3	1.5	0.5	0.3	56.88
Hugo	Male	Terrier	2	1	Relevant	Vocal	Irrelevant, Silent-Vocal	1	0.7	0	1.6	31.46
Hugo	Male	Terrier	2	2	Relevant	Vocal	Irrelevant, Silent-Vocal	1	4.5	0	0	16.62
Hugo	Male	Terrier	2	3	Relevant	Vocal	Irrelevant, Silent-Vocal	0	6.2	0	1.6	35.76
Hugo	Male	Terrier	2	4	Relevant	Silent	Irrelevant, Silent-Vocal	0	0	0	1.55	15.32
Hugo	Male	Terrier	2	5	Relevant	Silent	Irrelevant, Silent-Vocal	0	5.7	0	4.1	20.60
Hugo	Male	Terrier	2	6	Relevant	Silent	Irrelevant, Silent-Vocal	0	12.6	0	3.2	15.77
Isabelle	Female	Working	2	1	Relevant	Vocal	Relevant, Vocal-Silent	1	0.5	0	0	21.62
Isabelle	Female	Working	2	2	Relevant	Vocal	Relevant, Vocal-Silent	3	2.4	0	1.75	35.00
Isabelle	Female	Working	2	3	Relevant	Vocal	Relevant, Vocal-Silent	1	0.9	0.5	1.05	19.06
Isabelle	Female	Working	2	4	Relevant	Silent	Relevant, Vocal-Silent	0	0	0.5	0.85	17.50
Isabelle	Female	Working	2	5	Relevant	Silent	Relevant, Vocal-Silent	0	0	0	0.25	15.90
Isabelle	Female	Working	2	6	Relevant	Silent	Relevant, Vocal-Silent	1	0.5	0	0	22.37
Jago	Male	Working	5	1	Irrelevant	Silent	Irrelevant, Silent-Vocal	1	1.6	0	1	25.17
Jago	Male	Working	5	2	Irrelevant	Silent	Irrelevant, Silent-Vocal	0	2	0	1.85	25.08
Jago	Male	Working	5	3	Irrelevant	Silent	Irrelevant, Silent-Vocal	0	0	0	2.65	23.55
Jago	Male	Working	5	4	Irrelevant	Vocal	Irrelevant, Silent-Vocal	0	1.2	0	0	2.80
Jago	Male	Working	5	5	Irrelevant	Vocal	Irrelevant, Silent-Vocal	0	1.3	0	0	13.97
Jago	Male	Working	5	6	Irrelevant	Vocal	Irrelevant, Silent-Vocal	3	2.2	0	3.2	17.55
Jango	Male	Working	10	1	Relevant	Silent	Relevant, Silent-Vocal	1	0.5	0	4.75	14.92
Jango	Male	Working	10	2	Relevant	Silent	Relevant, Silent-Vocal	1	0.5	1.5	1.3	46.88
Jango	Male	Working	10	3	Relevant	Silent	Relevant, Silent-Vocal	0	0	1.5	1.4	9.71
Jango	Male	Working	10	4	Relevant	Vocal	Relevant, Silent-Vocal	0	3.3	1.5	2.6	17.92
Jango	Male	Working	10	5	Relevant	Vocal	Relevant, Silent-Vocal	1	1.7	1	4.6	13.33
Jango	Male	Working	10	6	Relevant	Vocal	Relevant, Silent-Vocal	3	2.8	0.5	0.8	23.89
Kip	Female	Pastoral	5.5	1	Relevant	Silent	Relevant, Silent-Vocal	0	0.9	0	0	13.00
Kip	Female	Pastoral	5.5	2	Relevant	Silent	Relevant, Silent-Vocal	0	1.1	0	0	28.06
Kip	Female	Pastoral	5.5	3	Relevant	Silent	Relevant, Silent-Vocal	0	0	0	7.05	3.51
Kip	Female	Pastoral	5.5	4	Relevant	Vocal	Relevant, Silent-Vocal	1	0.5	3	1.25	5.08
Kip	Female	Pastoral	5.5	5	Relevant	Vocal	Relevant, Silent-Vocal	1	10.5	0	0	9.94
Kip	Female	Pastoral	5.5	6	Relevant	Vocal	Relevant, Silent-Vocal	0	0	0.5	1.55	1.83
Kite	Female	Pastoral	3.5	1	Irrelevant	Silent	Relevant, Vocal-Silent	1	0.8	0	2.5	70.98
Kite	Female	Pastoral	3.5	2	Irrelevant	Silent	Relevant, Vocal-Silent	0	1.8	0	0.55	14.86
Kite	Female	Pastoral	3.5	3	Irrelevant	Silent	Relevant, Vocal-Silent	0	2.4	0	1.35	17.52
Kite	Female	Pastoral	3.5	4	Irrelevant	Vocal	Relevant, Vocal-Silent	0	1.6	0	0	27.59
Kite	Female	Pastoral	3.5	5	Irrelevant	Vocal	Relevant, Vocal-Silent	0	0.5	0	0	23.67
Kite	Female	Pastoral	3.5	6	Irrelevant	Vocal	Relevant, Vocal-Silent	0	0	0	2.3	21.45
Krug	Male	Gundog	4	1	Irrelevant	Silent	Irrelevant, Silent-Vocal	0	0	0.5	1.35	33.05
Krug	Male	Gundog	4	2	Irrelevant	Silent	Irrelevant, Silent-Vocal	1	1.5	0	1.25	31.12
Krug	Male	Gundog	4	3	Irrelevant	Silent	Irrelevant, Silent-Vocal	0	0.8	1	1.5	33.01
Krug	Male	Gundog	4	4	Irrelevant	Vocal	Irrelevant, Silent-Vocal	0	0	0.5	0.25	21.80
Krug	Male	Gundog	4	5	Irrelevant	Vocal	Irrelevant, Silent-Vocal	1	0.5	1	0.75	48.86
Krug	Male	Gundog	4	6	Irrelevant	Vocal	Irrelevant, Silent-Vocal	0	0	0.5	0.6	14.24
Lexi	Female	Working	1.5	1	Irrelevant	Vocal	Irrelevant, Vocal-Silent	0	0	1.5	2.7	70.79
Lexi	Female	Working	1.5	2	Irrelevant	Vocal	Irrelevant, Vocal-Silent	0	0	0	0	63.95
Lexi	Female	Working	1.5	3	Irrelevant	Vocal	Irrelevant, Vocal-Silent	0	0	0	0.45	52.50
Lexi	Female	Working	1.5	4	Irrelevant	Silent	Irrelevant, Vocal-Silent	1	1.7	1	3.85	60.26
Lexi	Female	Working	1.5	5	Irrelevant	Silent	Irrelevant, Vocal-Silent	0	0	1	2.6	35.06
Lexi	Female	Working	1.5	6	Irrelevant	Silent	Irrelevant, Vocal-Silent	0	1.3	0.5	7.05	21.08
Lola	Female	Cross	4	1	Irrelevant	Silent	Irrelevant, Silent-Vocal	0	0.5	0	0	16.23
Lola	Female	Cross	4	2	Irrelevant	Silent	Irrelevant, Silent-Vocal	0	0	1	0.55	10.54
Lola	Female	Cross	4	3	Irrelevant	Silent	Irrelevant, Silent-Vocal	1	0.9	0.5	1.4	21.19
Lola	Female	Cross	4	4	Irrelevant	Vocal	Irrelevant, Silent-Vocal	0	0	0.5	0.85	11.32
Lola	Female	Cross	4	5	Irrelevant	Vocal	Irrelevant, Silent-Vocal	0	1.2	1	0.35	28.44
Lola	Female	Cross	4	6	Irrelevant	Vocal	Irrelevant, Silent-Vocal	0	0.9	1	1.5	13.17
LolaJ	Female	Cross	1.5	1	Irrelevant	Vocal	Irrelevant, Vocal-Silent	0	0	0	0.6	9.87
LolaJ	Female	Cross	1.5	2	Irrelevant	Vocal	Irrelevant, Vocal-Silent	2	1.8	0	0	14.42
LolaJ	Female	Cross	1.5	3	Irrelevant	Vocal	Irrelevant, Vocal-Silent	0	1	0	0.4	11.43
LolaJ	Female	Cross	1.5	4	Irrelevant	Silent	Irrelevant, Vocal-Silent	1	4.5	0	1.15	7.67
LolaJ	Female	Cross	1.5	5	Irrelevant	Silent	Irrelevant, Vocal-Silent	0	0	2	0.5	3.07
LolaJ	Female	Cross	1.5	6	Irrelevant	Silent	Irrelevant, Vocal-Silent	0	0	0	0.3	1.80
Macey	Female	Gundog	6.5	1	Irrelevant	Vocal	Irrelevant, Vocal-Silent	3	1.8	0	0	49.84
Macey	Female	Gundog	6.5	2	Irrelevant	Vocal	Irrelevant, Vocal-Silent	0	2.2	0	0	22.09
Macey	Female	Gundog	6.5	3	Irrelevant	Vocal	Irrelevant, Vocal-Silent	2	4.5	0.5	0.5	13.71
Macey	Female	Gundog	6.5	4	Irrelevant	Silent	Irrelevant, Vocal-Silent	1	0.6	0	0	15.72
Macey	Female	Gundog	6.5	5	Irrelevant	Silent	Irrelevant, Vocal-Silent	0	1.5	0	0	15.76
Macey	Female	Gundog	6.5	6	Irrelevant	Silent	Irrelevant, Vocal-Silent	0	0	0	1.8	7.52
Marcus	Male	Gundog	8.5	1	Relevant	Vocal	Relevant, Vocal-Silent	0	1.4	0	0	41.78
Marcus	Male	Gundog	8.5	2	Relevant	Vocal	Relevant, Vocal-Silent	0	2.6	0	5.05	24.20
Marcus	Male	Gundog	8.5	3	Relevant	Vocal	Relevant, Vocal-Silent	0	0	0	0	24.03
Marcus	Male	Gundog	8.5	4	Relevant	Silent	Relevant, Vocal-Silent	2	1.8	0	0	38.36
Marcus	Male	Gundog	8.5	5	Relevant	Silent	Relevant, Vocal-Silent	0	6	0.5	2.8	18.92
Marcus	Male	Gundog	8.5	6	Relevant	Silent	Relevant, Vocal-Silent	0	10.7	0.5	1.5	15.34
Max	Male	Utility	4	1	Irrelevant	Vocal	Irrelevant, Vocal-Silent	0	0	2	1.3	31.35
Max	Male	Utility	4	2	Irrelevant	Vocal	Irrelevant, Vocal-Silent	2	2.1	2	1.35	9.87
Max	Male	Utility	4	3	Irrelevant	Vocal	Irrelevant, Vocal-Silent	2	2.5	0	0	9.87
Max	Male	Utility	4	4	Irrelevant	Silent	Irrelevant, Vocal-Silent	1	2.8	0.5	1.5	14.75
Max	Male	Utility	4	5	Irrelevant	Silent	Irrelevant, Vocal-Silent	2	5.1	0	0	11.69
Max	Male	Utility	4	6	Irrelevant	Silent	Irrelevant, Vocal-Silent	2	4	0	0	11.88
Merlin	Male	Gundog	8.5	1	Irrelevant	Vocal	Irrelevant, Vocal-Silent	0	0	0	0	18.63
Merlin	Male	Gundog	8.5	2	Irrelevant	Vocal	Irrelevant, Vocal-Silent	1	0.5	0	4.25	11.70
Merlin	Male	Gundog	8.5	3	Irrelevant	Vocal	Irrelevant, Vocal-Silent	0	0	0	6.8	8.33
Merlin	Male	Gundog	8.5	4	Irrelevant	Silent	Irrelevant, Vocal-Silent	1	3	0	7.4	6.73
Merlin	Male	Gundog	8.5	5	Irrelevant	Silent	Irrelevant, Vocal-Silent	2	14.6	0	0.3	16.56
Merlin	Male	Gundog	8.5	6	Irrelevant	Silent	Irrelevant, Vocal-Silent	0	0	0	7.75	12.98
MerlinY	Male	Gundog	3.5	1	Relevant	Vocal	Relevant, Vocal-Silent	1	2.7	0.5	0.35	63.88
MerlinY	Male	Gundog	3.5	2	Relevant	Vocal	Relevant, Vocal-Silent	0	0	0.5	1.95	4.52
MerlinY	Male	Gundog	3.5	3	Relevant	Vocal	Relevant, Vocal-Silent	3	1.8	0	0	12.42
MerlinY	Male	Gundog	3.5	4	Relevant	Silent	Relevant, Vocal-Silent	0	5.5	0	0	13.61
MerlinY	Male	Gundog	3.5	5	Relevant	Silent	Relevant, Vocal-Silent	1	6.1	0.5	1.6	23.29
MerlinY	Male	Gundog	3.5	6	Relevant	Silent	Relevant, Vocal-Silent	2	2.7	0	0.65	23.30
Mishka	Female	Working	1.5	1	Irrelevant	Vocal	Irrelevant, Vocal-Silent	1	1.4	1	1.35	11.61
Mishka	Female	Working	1.5	2	Irrelevant	Vocal	Irrelevant, Vocal-Silent	1	0.7	0	0	42.09
Mishka	Female	Working	1.5	3	Irrelevant	Vocal	Irrelevant, Vocal-Silent	0	0	0	0.65	32.36
Mishka	Female	Working	1.5	4	Irrelevant	Silent	Irrelevant, Vocal-Silent	0	0	1.5	1.2	28.66
Mishka	Female	Working	1.5	5	Irrelevant	Silent	Irrelevant, Vocal-Silent	0	0	0.5	0.85	18.15
Mishka	Female	Working	1.5	6	Irrelevant	Silent	Irrelevant, Vocal-Silent	1	9	0	0.25	28.62
Misty	Female	Cross	10	1	Relevant	Vocal	Relevant, Vocal-Silent	1	9.1	0	0	12.20
Misty	Female	Cross	10	2	Relevant	Vocal	Relevant, Vocal-Silent	0	0	0	4.55	13.08
Misty	Female	Cross	10	3	Relevant	Vocal	Relevant, Vocal-Silent	0	1.6	0	1.05	20.90
Misty	Female	Cross	10	4	Relevant	Silent	Relevant, Vocal-Silent	0	13.1	0	0.3	7.37
Misty	Female	Cross	10	5	Relevant	Silent	Relevant, Vocal-Silent	0	0	0	0	11.29
Misty	Female	Cross	10	6	Relevant	Silent	Relevant, Vocal-Silent	1	0.5	0	0	21.96
Monty	Male	Gundog	2	1	Relevant	Silent	Relevant, Silent-Vocal	4	1.3	2	0.95	22.86
Monty	Male	Gundog	2	2	Relevant	Silent	Relevant, Silent-Vocal	2	2	0	0	26.02
Monty	Male	Gundog	2	3	Relevant	Silent	Relevant, Silent-Vocal	0	0	0	0	15.41
Monty	Male	Gundog	2	4	Relevant	Vocal	Relevant, Silent-Vocal	0	2.3	0.5	2.3	12.99
Monty	Male	Gundog	2	5	Relevant	Vocal	Relevant, Silent-Vocal	0	0	0.5	4.3	41.67
Monty	Male	Gundog	2	6	Relevant	Vocal	Relevant, Silent-Vocal	0	0.5	0	0	8.97
MontyS	Male	Utility	5	1	Irrelevant	Vocal	Irrelevant, Vocal-Silent	1	2.4	0.5	0.45	31.48
MontyS	Male	Utility	5	2	Irrelevant	Vocal	Irrelevant, Vocal-Silent	0	0	0.5	0.75	56.49
MontyS	Male	Utility	5	3	Irrelevant	Vocal	Irrelevant, Vocal-Silent	2	1.3	0	0	31.01

MontyS	Male	Utility	5	4	Irrelevant	Silent	Irrelevant, Vocal-Silent	0	0.5	1	0.25	32.28
MontyS	Male	Utility	5	5	Irrelevant	Silent	Irrelevant, Vocal-Silent	0	0	0	0	30.37
MontyS	Male	Utility	5	6	Irrelevant	Silent	Irrelevant, Vocal-Silent	0	0	0	0	24.44
Murphy	Male	Cross	6	1	Relevant	Silent	Relevant, Silent-Vocal	0	0	2.5	4.45	17.16
Murphy	Male	Cross	6	2	Relevant	Silent	Relevant, Silent-Vocal	4	7.7	2.5	5.35	11.39
Murphy	Male	Cross	6	3	Relevant	Silent	Relevant, Silent-Vocal	1	1.8	1	0.45	9.12
Murphy	Male	Cross	6	4	Relevant	Vocal	Relevant, Silent-Vocal	0	0	0	0	13.75
Murphy	Male	Cross	6	5	Relevant	Vocal	Relevant, Silent-Vocal	1	0.5	0	0	3.77
Murphy	Male	Cross	6	6	Relevant	Vocal	Relevant, Silent-Vocal	0	0	0	0	18.63
Nelson	Male	Gundog	9.5	1	Relevant	Silent	Relevant, Silent-Vocal	0	0	0	0	33.20
Nelson	Male	Gundog	9.5	2	Relevant	Silent	Relevant, Silent-Vocal	0	11.1	0	0	21.98
Nelson	Male	Gundog	9.5	3	Relevant	Silent	Relevant, Silent-Vocal	0	1.1	0	0	23.22
Nelson	Male	Gundog	9.5	4	Relevant	Vocal	Relevant, Silent-Vocal	0	0	0	1.55	21.42
Nelson	Male	Gundog	9.5	5	Relevant	Vocal	Relevant, Silent-Vocal	0	0	0	0	16.48
Nelson	Male	Gundog	9.5	6	Relevant	Vocal	Relevant, Silent-Vocal	0	0	0	0	11.56
Oppo	Male	Gundog	3.5	1	Irrelevant	Silent	Irrelevant, Silent-Vocal	0	0	1	2.3	68.05
Oppo	Male	Gundog	3.5	2	Irrelevant	Silent	Irrelevant, Silent-Vocal	3	1.3	0	0	28.48
Oppo	Male	Gundog	3.5	3	Irrelevant	Silent	Irrelevant, Silent-Vocal	1	0.5	0	0	46.96
Oppo	Male	Gundog	3.5	4	Irrelevant	Vocal	Irrelevant, Silent-Vocal	1	0.5	0	0.7	30.19
Oppo	Male	Gundog	3.5	5	Irrelevant	Vocal	Irrelevant, Silent-Vocal	0	0	0	0	26.93
Oppo	Male	Gundog	3.5	6	Irrelevant	Vocal	Irrelevant, Silent-Vocal	2	0.8	0	0	45.51
Oscar	Male	Cross	1	1	Relevant	Silent	Relevant, Silent-Vocal	0	0	0.5	0.5	65.06
Oscar	Male	Cross	1	2	Relevant	Silent	Relevant, Silent-Vocal	0	0	2	0.7	47.85
Oscar	Male	Cross	1	3	Relevant	Silent	Relevant, Silent-Vocal	2	3.7	0	0	56.88
Oscar	Male	Cross	1	4	Relevant	Vocal	Relevant, Silent-Vocal	0	0	0	0	50.32
Oscar	Male	Cross	1	5	Relevant	Vocal	Relevant, Silent-Vocal	4	4.2	0	0	28.25
Oscar	Male	Cross	1	6	Relevant	Vocal	Relevant, Silent-Vocal	2	2.6	0	0	47.57
Ozzie	Male	Pastoral	6	1	Relevant	Silent	Relevant, Silent-Vocal	0	0	0	1.65	81.79
Ozzie	Male	Pastoral	6	2	Relevant	Silent	Relevant, Silent-Vocal	0	0.6	1	0.25	20.90
Ozzie	Male	Pastoral	6	3	Relevant	Silent	Relevant, Silent-Vocal	5	2.3	0	0	31.71
Ozzie	Male	Pastoral	6	4	Relevant	Vocal	Relevant, Silent-Vocal	2	2.5	1	3.15	10.66
Ozzie	Male	Pastoral	6	5	Relevant	Vocal	Relevant, Silent-Vocal	0	0	0	0	18.35
Ozzie	Male	Pastoral	6	6	Relevant	Vocal	Relevant, Silent-Vocal	1	1	0	0	35.00
Pippa	Female	Terrier	5	1	Irrelevant	Silent	Irrelevant, Silent-Vocal	1	0.9	0.5	2.15	10.44
Pippa	Female	Terrier	5	2	Irrelevant	Silent	Irrelevant, Silent-Vocal	3	7.4	0	0	8.02
Pippa	Female	Terrier	5	3	Irrelevant	Silent	Irrelevant, Silent-Vocal	0	0	0	0	1.52
Pippa	Female	Terrier	5	4	Irrelevant	Vocal	Irrelevant, Silent-Vocal	5	3.4	0.5	1.1	8.24
Pippa	Female	Terrier	5	5	Irrelevant	Vocal	Irrelevant, Silent-Vocal	0	0	0	0	1.89
Pippa	Female	Terrier	5	6	Irrelevant	Vocal	Irrelevant, Silent-Vocal	0	0	0	0	11.11
Quito	Male	Pastoral	6	1	Irrelevant	Silent	Irrelevant, Silent-Vocal	0	0	0.5	0.25	39.56
Quito	Male	Pastoral	6	2	Irrelevant	Silent	Irrelevant, Silent-Vocal	0	0	0.5	0.6	28.48
Quito	Male	Pastoral	6	3	Irrelevant	Silent	Irrelevant, Silent-Vocal	0	0	0	0.3	5.52
Quito	Male	Pastoral	6	4	Irrelevant	Vocal	Irrelevant, Silent-Vocal	0	0	1	0.65	13.23
Quito	Male	Pastoral	6	5	Irrelevant	Vocal	Irrelevant, Silent-Vocal	0	0	0.5	0.25	29.47
Quito	Male	Pastoral	6	6	Irrelevant	Vocal	Irrelevant, Silent-Vocal	1	0.5	1	0.55	17.72
Skye	Female	Cross	2	1	Relevant	Silent	Relevant, Silent-Vocal	0	1.1	1.5	1.95	17.82
Skye	Female	Cross	2	2	Relevant	Silent	Relevant, Silent-Vocal	2	4.6	1.5	6	16.61
Skye	Female	Cross	2	3	Relevant	Silent	Relevant, Silent-Vocal	1	0.6	0.5	1.8	6.69
Skye	Female	Cross	2	4	Relevant	Vocal	Relevant, Silent-Vocal	0	0	1	3.2	3.28
Skye	Female	Cross	2	5	Relevant	Vocal	Relevant, Silent-Vocal	0	0	1	1.05	9.97
Skye	Female	Cross	2	6	Relevant	Vocal	Relevant, Silent-Vocal	1	3.7	0	0.3	11.48
Snoopy	Male	Cross	2	1	Irrelevant	Silent	Irrelevant, Silent-Vocal	0	0	0	0	5.83
Snoopy	Male	Cross	2	2	Irrelevant	Silent	Irrelevant, Silent-Vocal	0	2.1	0	0	30.28
Snoopy	Male	Cross	2	3	Irrelevant	Silent	Irrelevant, Silent-Vocal	0	0	0.5	6.4	18.91
Snoopy	Male	Cross	2	4	Irrelevant	Vocal	Irrelevant, Silent-Vocal	0	0	0	0	8.70
Snoopy	Male	Cross	2	5	Irrelevant	Vocal	Irrelevant, Silent-Vocal	0	0	0	0	12.62
Snoopy	Male	Cross	2	6	Irrelevant	Vocal	Irrelevant, Silent-Vocal	2	1.4	0	0	89.35
Toby	Male	Gundog	4.5	1	Relevant	Vocal	Relevant, Vocal-Silent	2	1.4	0	0	26.18
Toby	Male	Gundog	4.5	2	Relevant	Vocal	Relevant, Vocal-Silent	0	0	0.5	0.95	6.89
Toby	Male	Gundog	4.5	3	Relevant	Vocal	Relevant, Vocal-Silent	0	0	2	2.6	11.33
Toby	Male	Gundog	4.5	4	Relevant	Silent	Relevant, Vocal-Silent	1	1.2	0	0	6.15
Toby	Male	Gundog	4.5	5	Relevant	Silent	Relevant, Vocal-Silent	2	1.1	0	0.8	26.80
Toby	Male	Gundog	4.5	6	Relevant	Silent	Relevant, Vocal-Silent	0	0	1.5	3.3	8.49
Tubby	Male	Toy	3.5	1	Relevant	Vocal	Relevant, Vocal-Silent	0	0.5	0	0	33.54
Tubby	Male	Toy	3.5	2	Relevant	Vocal	Relevant, Vocal-Silent	0	2.2	0	0	14.74
Tubby	Male	Toy	3.5	3	Relevant	Vocal	Relevant, Vocal-Silent	0	1	0	0.35	23.12
Tubby	Male	Toy	3.5	4	Relevant	Silent	Relevant, Vocal-Silent	1	8.5	0	0	26.45
Tubby	Male	Toy	3.5	5	Relevant	Silent	Relevant, Vocal-Silent	0	0	0.5	0.65	51.80
Tubby	Male	Toy	3.5	6	Relevant	Silent	Relevant, Vocal-Silent	0	0	0	0	10.78
Whilma	Female	Hound	3	1	Relevant	Vocal	Relevant, Vocal-Silent	0	4.2	0	0	8.16
Whilma	Female	Hound	3	2	Relevant	Vocal	Relevant, Vocal-Silent	0	1	1	3.85	20.66
Whilma	Female	Hound	3	3	Relevant	Vocal	Relevant, Vocal-Silent	3	1	0	2.35	11.94
Whilma	Female	Hound	3	4	Relevant	Silent	Relevant, Vocal-Silent	0	0.6	0	1.4	6.62
Whilma	Female	Hound	3	5	Relevant	Silent	Relevant, Vocal-Silent	3	5.2	0.5	0.65	8.33
Whilma	Female	Hound	3	6	Relevant	Silent	Relevant, Vocal-Silent	2	1	0	0	1.66
Wilson	Male	Cross	1.5	1	Relevant	Vocal	Relevant, Vocal-Silent	0	0	0.5	1.95	37.23
Wilson	Male	Cross	1.5	2	Relevant	Vocal	Relevant, Vocal-Silent	0	0.5	0	1.55	21.35
Wilson	Male	Cross	1.5	3	Relevant	Vocal	Relevant, Vocal-Silent	0	0	0	0.8	36.70
Wilson	Male	Cross	1.5	4	Relevant	Silent	Relevant, Vocal-Silent	0	0	1	4.5	21.18
Wilson	Male	Cross	1.5	5	Relevant	Silent	Relevant, Vocal-Silent	0	0	0	0.65	14.33
Wilson	Male	Cross	1.5	6	Relevant	Silent	Relevant, Vocal-Silent	0	0	0	0.3	17.09
Zippy	Male	Cross	3	1	Relevant	Silent	Relevant, Silent-Vocal	0	0.8	1	0.5	28.39
Zippy	Male	Cross	3	2	Relevant	Silent	Relevant, Silent-Vocal	0	2	0.5	1.15	17.52
Zippy	Male	Cross	3	3	Relevant	Silent	Relevant, Silent-Vocal	0	0	0	1.2	19.03
Zippy	Male	Cross	3	4	Relevant	Vocal	Relevant, Silent-Vocal	1	1.1	0	0.7	16.35
Zippy	Male	Cross	3	5	Relevant	Vocal	Relevant, Silent-Vocal	3	3.7	0.5	0.35	16.72
Zippy	Male	Cross	3	6	Relevant	Vocal	Relevant, Silent-Vocal	0	0	1.5	1.65	16.14
