# Supplementary material for: Do Dogs Provide Information Helpfully?
Source: PLoS One. 2016 Aug 10;11(8):e0159797. doi: 10.1371/journal.pone.0159797 (PMC4980001; doi:10.1371/journal.pone.0159797)
Supplement: S1 Text — (PDF) [file pone.0159797.s002.pdf]

Statistical Analysis: model fitting additional information

Study 1

For the response variable "indication of the target" we were interested in the effect of the experimenter's utterance during the search in each condition. Therefore the global model was calculated adding the fixed factors "utterance" (before utterance and after utterance) and "condition" (*relevant object, useless object, no object*) with an interaction. The fixed factor "attention during the demonstration" (i.e. percentage of time spent looking at the experimenter in the demonstration phase) was added as additional interaction with the previous factors, because it was expected that dogs' attention affected the indications of the target differently based on the condition. The fixed factors "gender" (male and female) and "trial number" (1 to 6) were also added, without interaction, to control for their main effect on the response.

For the response variable "frequency of gaze alternations" we were interested in the effect of the content of the target box on gaze alternations. Therefore the global model was calculated adding the fixed factor "condition" (*relevant object, useless object, no object*) to the null model. To control for the effect of dogs' attention, the fixed factor "attention during the demonstration" (i.e. percentage of time spent looking at the experimenter in the demonstration phase) was added to the model. Because it was expected that the frequency of gaze alternations differed based on their direction and across conditions, and that dogs' attention affected the gaze alternations to the target differently based on the condition, the factors "direction", "condition" and "attention" were included in the model with a 3 level interaction. The fixed

factors “gender” (male and female) and “trial number” (1 to 6) were also added, without interaction, to control for their main effect on the response.

For the response variable “duration of gazes (s)” we were again interested in the effect of the content of the target box on dogs’ looking behaviour. Therefore we calculated the global model adding the fixed factor “condition” (content of the target, i.e. *relevant object, useless object, no object*) to the null model. To control for the effect of dogs’ attention, the fixed factor “attention during the demonstration” (i.e. percentage of time spent looking at the experimenter in the demonstration phase) was added to the model. It was expected that the duration of gazes varied based on their direction, and that dogs’ attention affected dog gazes to the target differently based on the condition. Therefore the factors “direction”, “condition” and “attention” had a 3 level interaction. The fixed factors “gender” (male and female) and “trial number” (1 to 6) were also added to the model, without interaction, to control for their main effect on the response.

Study 2

For the response variable “gaze alternations” (number of gaze alternations toward the target box) we were interested in the effect of communication style and the object hidden in the target box, therefore a global model was calculated adding the fixed factors “communication” (silent or vocal) and “condition” (*relevant* group or *distractor* group). We expected the “attention” during the demonstration (i.e. percentage of time spent looking at the experimenter) to affect the frequency of gaze alternations therefore we added the fixed factor “attention” to the global model. We

expected the communication style and the attention during the demonstration to affect differently the dogs in the relevant group and those in the random group, so a 3 levels interaction between “communication”, “condition” and “attention” was included. The fixed factors “gender” (male and female) and “trial number” (1 to 6) were also added, without interaction, to control for their main effect on the response.

For the response variable “duration of gazes (s)” we calculated a global model by adding the fixed factor “direction” (direction of the gaze alternation, i.e. target box or empty box) to the null model in order to allow assessing the effects of the other factors on different directions of gazes (i.e. empty boxes and target box). We were interested in the effect of communication style and the object hidden in the target box, therefore a global model was calculated adding the fixed factors “communication” (silent or vocal) and “condition” (*relevant* group or *distractor* group). In order investigate the different effects of communication on the duration of gazes in the two conditions and when looking at different boxes, the factors “direction”, “condition”, and “communication” were included in the global model with a 3 level interaction. Following the results of study 1, we expected the “attention” during the demonstration (i.e. percentage of time spent looking at the experimenter) to have a main effect on the persistency of gazes therefore we added the fixed factor “attention” to the global model. The fixed factors “gender” (male and female) and “trial number” (1 to 6) were also added, without interaction, to control for their main effect on the response
